# International Prognostic Index-Based Immune Prognostic Model for Diffuse Large B-Cell Lymphoma

**DOI:** 10.3389/fimmu.2021.732006

**Published:** 2021-10-22

**Authors:** Shidai Mu, Deyao Shi, Lisha Ai, Fengjuan Fan, Fei Peng, Chunyan Sun, Yu Hu

**Affiliations:** ^1^ Institution of Hematology, Union Hospital, Tongji Medical College, Huazhong University of Science and Technology, Wuhan, China; ^2^ Department of Orthopaedics, Union Hospital, Tongji Medical College, Huazhong University of Science and Technology, Wuhan, China

**Keywords:** diffuse large B-cell lymphoma, immune prognostic model, nomogram, immunotherapy, tumor microenvironment

## Abstract

**Background:**

The International Prognostic Index (IPI) is widely used to discriminate the prognosis of patients with diffuse large B-cell lymphoma (DLBCL). However, there is a significant need to identify novel valuable biomarkers in the context of targeted therapy, such as immune checkpoint blockade (ICB).

**Methods:**

Gene expression data and clinical DLBCL information were obtained from The Cancer Genome Atlas and Gene Expression Omnibus datasets. A total of 371 immune-related genes in DLBCL patients associated with different IPI risk groups were identified by weighted gene co-expression network analysis, and eight genes were selected to construct an IPI-based immune prognostic model (IPI-IPM). Subsequently, we analyzed the somatic mutation and transcription profiles of the IPI-IPM subgroups as well as the potential clinical response to immune checkpoint blockade (ICB) in IPI-IPM subgroups.

**Results:**

The IPI-IPM was constructed based on the expression of *CMBL*, *TLCD3B*, *SYNDIG1*, *ESM1*, *EPHA3*, *HUNK*, *PTX3*, and *IL12A*, where high-risk patients had worse overall survival than low-risk patients, consistent with the results in the independent validation cohorts. The comprehensive results showed that high IPI-IPM risk scores were correlated with immune-related signaling pathways, high *KMT2D* and *CD79B* mutation rates, and upregulation of inhibitory immune checkpoints, including *PD-L1*, *BTLA*, and *SIGLEC7*, indicating a greater potential response to ICB therapy.

**Conclusion:**

The IPI-IPM has independent prognostic significance for DLBCL patients, which provides an immunological perspective to elucidate the mechanisms of tumor progression and sheds light on the development of immunotherapy for DLBCL.

## Introduction

Diffuse large B-cell lymphoma (DLBCL) accounts for approximately 40% of non-Hodgkin B-cell lymphoma, with an annual incidence rate of over 100,000 cases worldwide (1, 2). Although the current frontline DLBCL therapy (the standard R-CHOP chemotherapy regimen) is associated with a high complete response rate of 70%–80%, 10%–15% of DLBCL patients are refractory, and almost 40% of patients experience relapse within 2–3 years after initial response (3, 4). With the development of high-throughput technologies, germinal center B-cell-like and activated B-cell-like DLBCL subtypes were identified by gene expression profiling based on cell-of-origin classification (5–7). More recently, several key cytogenetic alterations including mutations, somatic copy number alterations, and structural variants have been shown to classify distinct genetic subtypes within the cell-of-origin subgroups, providing insights into heterogeneous disease pathogenesis and candidate treatment targets (1, 3, 7–9). Several prognostic factors including cell-of-origin and the International Prognostic Index (IPI) have already been identified in the rituximab era, which still need further investigation in the context of targeted therapies (7, 9–12). Therefore, there is an urgent need to explore potential molecular mechanisms and identify key biomarkers and therapeutic targets.

Accumulating evidence has shed light on the prognostic role of the tumor microenvironment (TME) in immune checkpoint blockade therapy (ICB), which is mostly composed of a variety of immune cells (T, NK, and B cells as well as macrophages) and stroma (blood vessels and extracellular matrix [ECM]) (13–16). Kotlov et al. (11) characterized the DLBCL TME into four distinct microenvironment compositions including “germinal center-like” (GC), “mesenchymal” (MS), “inflammatory” (IN), and “depleted” (DP) form, which are associated with distinct clinical behavior and provide novel potential targets for innovative therapeutic interventions.

In this study, we identified immune-related hub genes in DLBCL patients at different IPI levels by weighted gene co-expression network analysis (WGCNA) and constructed an IPI-based immune-related prognostic model (IPI-IPM). We then characterized the somatic mutation and transcription profiles of the IPI-IPM subgroups, investigated the expression of several inhibitory immune checkpoints between low- and high-risk subgroups, and applied an unsupervised clustering algorithm to analyze the gene expression pattern of lymphoma microenvironment (LME) signatures. The results showed that IPI-IPM was a promising prognostic biomarker, which also has potential for use in patient management.

## Results

### Identification of Immune-Related Genes Associated With IPI in DLBCL Patients

**Figure 1:**
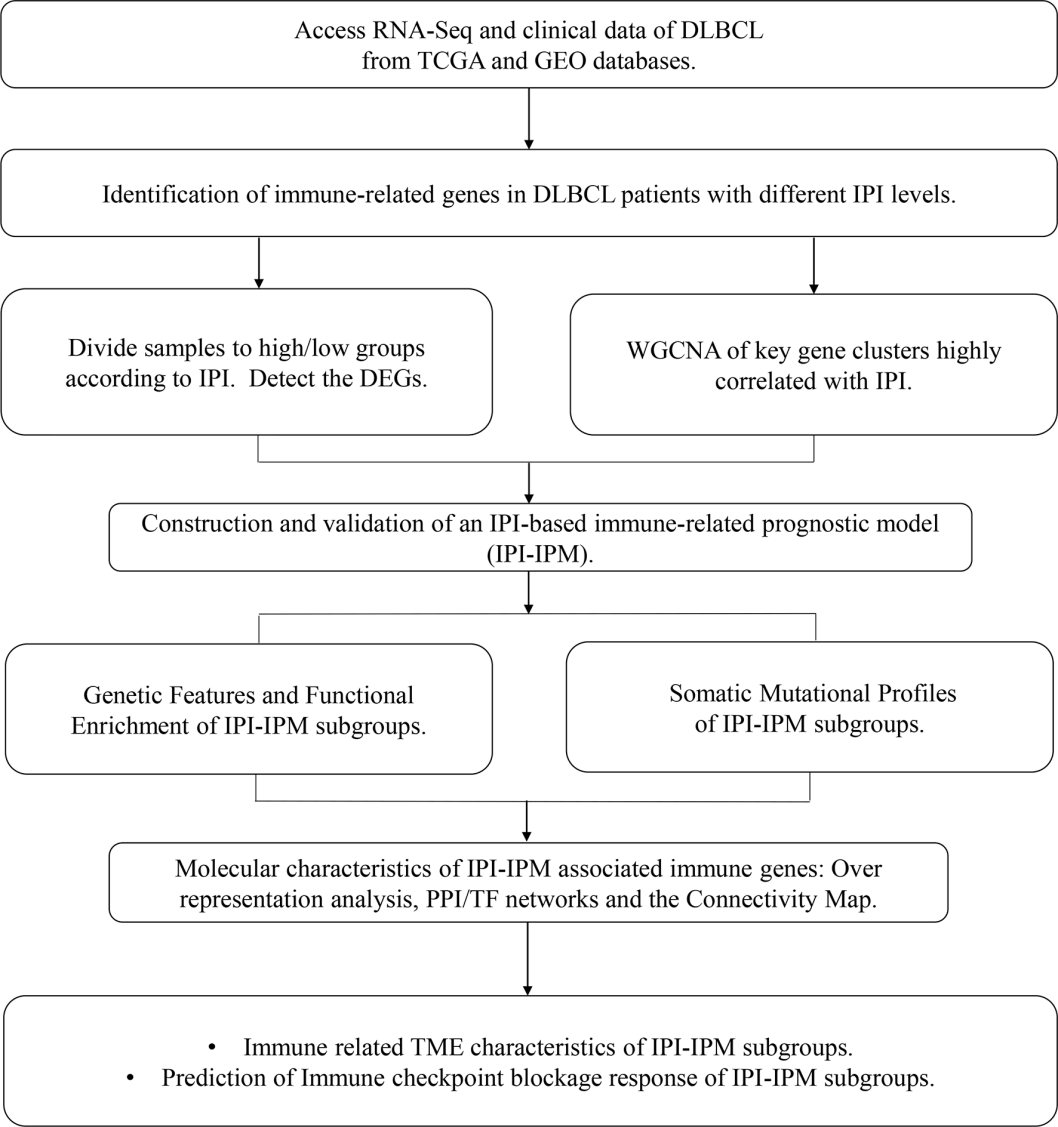
A flowchart for the process of the present study.

**Figure 2:**
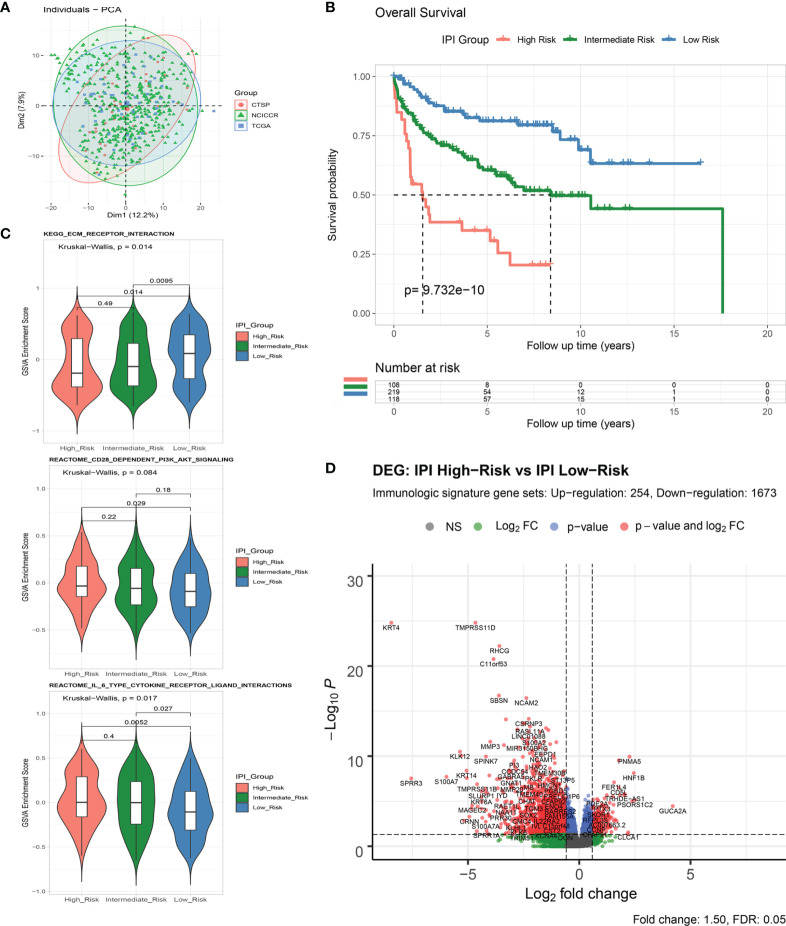
Analysis of immune-related genes in DLBCL patients of different IPI risk groups. **(A)** Principal component analysis of RNA-Seq count data from three included projects. **(B)** Survival analysis of overall survival between IPI Risk groups. **(C)** Gene set variance analysis (GSVA) of enriched gene sets between IPI Risk groups. **(D)** Volcano plot of differentially expressed immune-related genes (DEGs) between the high and low IPI risk groups.

A flowchart is shown to demonstrate the procedure and results of our study (**Figure 1**). RNA-seq data of 570 DLBCL patients were obtained from The Cancer Genome Atlas (TCGA) (48 from TCGA-DLBC, 41 from CTSP-DLBCL1, and 481 from NCICCR-DLBCL). Among the 566 DLBCL patients with overall survival (OS) data, 321 (56.71%) were male and 245 (43.29%) were female. The age of the patients ranged from 14 to 92 years (median, 62 years) at initial diagnosis. Other clinical characteristics, including follow-up period, Ann Arbor stages, lactate dehydrogenase (LDH) ratio, Eastern Cooperative Oncology Group (ECOG) performance status, and number of extranodal sites, are documented in **Table 1** and **Supplementary Material 1**. To remove the batch effect among these three projects, we utilized the ComBat-seq function to transform the raw count data using the sva R package. Then, principal component analysis was performed to show that there was no obvious batch effect among the samples (**Figure 2A**). After excluding samples with unrecorded IPI scores or with an IPI score crossing risk groups (such as 1–5 or 3–4), 458 DLBCL patients were divided into low-risk (n = 118), intermediate-risk (n = 221; 106 at low-intermediate risk, and 92 at high-intermediate risk), and high-risk groups (n = 109) (**Table 1**). Consistent with previous publications, patients in the low-IPI risk group had a much longer OS (**Figure 2B**).

**Table 1:** Clinical characteristics of 570 DLBCL patients from the TCGA.

Project			TCGA_DLBCL		NCICCR_DLBCL		CTSP_DLBCL1	
Gene expression data (RNA-seq)			48^①^		481		41	
Overall survival follow-up			48		481		37	
Age (range)			23~82		14~92^③^		27~84	
Gender	Female		26		195		24	
	Male		22		286		13	
IPI_group	Low risk		9		103		6	
	Intermediate risk	Low	13	5	185	88	23	13
		High		5		77		10
	High risk		3		98		8	
	NA^②^		23		95			

^①^Thirty-seven samples from TCGA_DLBCL with WES data.

^②^IPI range groups: 0–1 (low risk); 2–3 (intermediate risk); 2 (low-intermediate risk); 3 (high-intermediate risk); 4–5 (high risk). If the IPI is not recorded or the range of the IPI spans among groups (such as 1–5 or 1–2), this feature is marked as non-applicable (NA).

^③^Age of nine samples from NCICCR_DLBCL was not reported.

As shown by gene set variation analysis (GSVA), ECM receptor interaction, CD28-dependent PI3K/AKT signaling, IL-6-type cytokine receptor ligand interaction, and other immunologic signaling pathways were significantly enriched in the low-risk group (**Figure 2C** and **Supplementary Figures S1A, B**). A total of 4,651 genes (633 upregulated and 4,018 downregulated) were significantly differentially expressed between the IPI high- and low-risk groups (**Supplementary Figures S1C, D** and **Supplementary Material 2**). By intersecting with the immunologic signature gene sets (combining 20,837 genes from ImmuneSigDB and Immport, **Supplementary Material 3**), 1,927 immune differentially expressed genes (DEGs) (254 upregulated and 1,673 downregulated) were identified for further analysis (**Figure 2D** and **Supplementary Figures S1E, F**).

**Figure 3:**
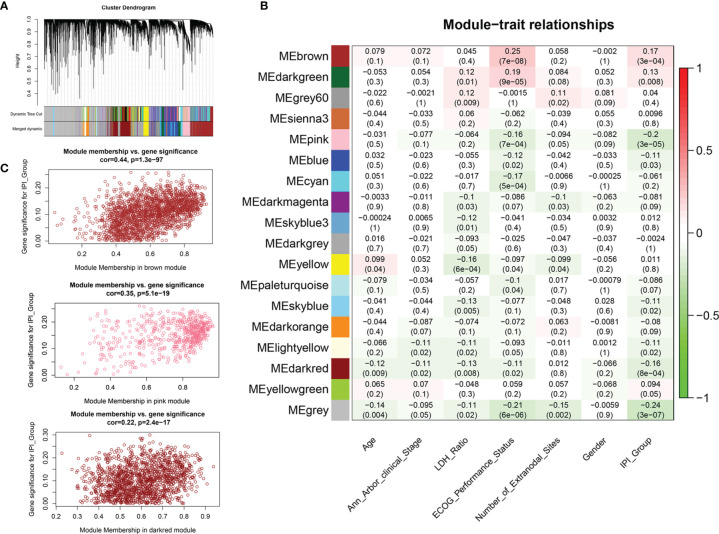
Weighted gene co-expression network analysis (WGCNA) for the identification of modules related to IPI risk group. **(A)** Process of clustering dendrogram of included genes, assigning module colors, and merging modules. **(B)** Analysis and visualization of Module-trait relationship to identify IPI risk group related modules. **(C)** Correlation between gene module membership and gene significance for IPI risk group in brown, pink, and dark-red modules.

We applied variance-stability-transformed (VST) expression data *via* DESeq2 as the input data for WGCNA, including 13,329 genes with the top 25% variance among all samples (**Figure 3A**). All clinical characteristics were enrolled as trait variables, and the best β value in the co-expression network was calculated to be 9 (**Supplementary Figure S2B**). The distance threshold for merging modules was set to be 0.30, so as to construct a reasonable number of merged modules (**Supplementary Figures S2A, D**). As shown in the module–trait relationship, eight modules were significantly correlated with the IPI group (**Figure 3B**), and a high correlation (p < 0.0001) between gene significance of IPI risk groups and gene module membership was found in the genes of three modules (brown, pink, dark red) (**Figure 3C** and **Supplementary Figures S2E, G**). By intersecting 4,106 genes from the top three IPI-correlating modules with 1,927 immune DEGs, a total of 371 genes were identified as immune-related genes associated with IPI, which were used for prognostic risk model construction (**Supplementary Figure S2H**). Several gene sets, such as ECM organization, ECM receptor interaction, PI3K/AKT signaling, and integrin cell surface interaction were enriched in the top three IPI-correlating modules (**Supplementary Figure S2I** and **Supplementary Material 4**).

### Construction and Validation of an IPI-Based Immune Prognostic Risk Model

**Figure 4:**
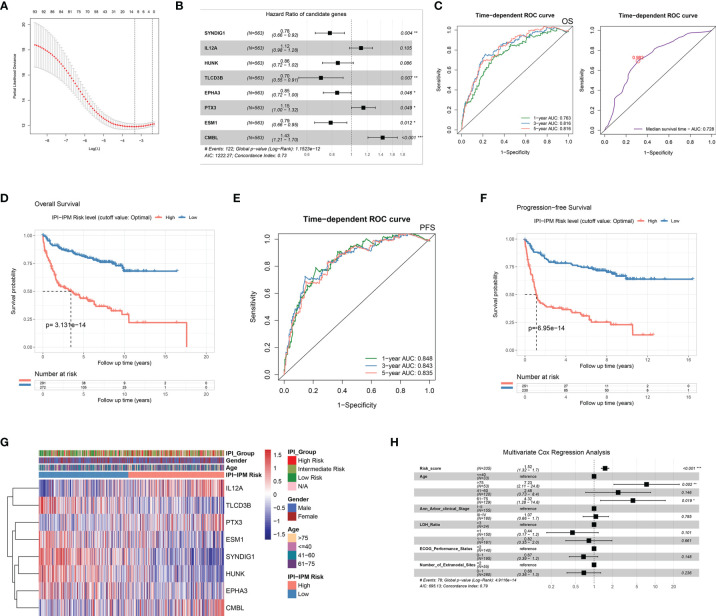
Construction of an IPI-based immune prognostic model. **(A)** A plot for displaying the cross-validation error according to the log of lambda in the Lasso penalized Cox regression. **(B)** A forest plot for hazard ratios of the eight genes composing the IPI-based immune prognostic model (IPI-IPM). **(C)** Time-dependent ROC curves for the IPI-IPM risk scores on overall survival. **(D)** Kaplan–Meier Survival analysis of overall survival for patients of high and low IPI-IPM risk groups. **(E)** Time-dependent ROC curves for the IPI-IPM risk scores on progression-free survival. **(F)** Kaplan–Meier survival analysis of progression-free survival for patients of high and low IPI-IPM risk groups. **(G)** Heatmap of the gene expression in high and low IPI-IPM risk groups. **(H)** The multivariate analysis of IPI-IPM risk score and clinicopathologic parameters including age, Ann Arbor clinical stage, LDH ratio, and ECOG performance status and the number of extranodal sites. P value, * < 0.05, ** < 0.01, *** < 0.001.

In the training cohort, of which 563 patients with matched RNA-seq data and overall survival follow up data, 93 out of 371 IPI-related immune genes were significantly correlated with OS in the univariate Cox regression analysis (**Table 2** and **Supplementary Material 5**). Next, we applied Lasso-penalized Cox regression to identify the optimal number of genes (n = 10) for the risk score model (**Figure 4A** and **Supplementary Figure S3A**). As a result of multivariate Cox regression analysis with variable selection by Akaike information criterion, eight genes were selected to construct the most optimal IPI-IPM (**Figure 4B**). Risk score = (expression level of *CMBL* * [0.360] + expression level of *TLCD3B* * [-0.350] + expression level of *SYNDIG1* * [-0.247] + expression level of *ESM1* * [-0.238] + expression level of *EPHA3* * [-0.163] + expression level of *HUNK* * [-0.156] + expression level of *PTX3* * [0.138] + expression level of *IL12A* * [0.111]). As shown in the time-dependent receiver operating characteristic (ROC) curve, areas under the curve (AUC) were 0.703, 0.738, and 0.733 for 1, 3, and 5 years, respectively (**Figure 4C**). As shown in **Supplementary Figure S3B**, there was a significant difference in OS and PFS between high- and low-risk groups, taking the median of IPI-IPM risk scores as the cutoff value. According to the ROC curve of the median survival time (**Supplementary Figure S3C**), we defined a cutoff value of 0.982 and then divided all patients into high- and low-risk groups according to their risk scores. As shown in **Figure 4D**, Kaplan–Meier survival analysis showed worse OS of patients in the high-risk score group (log-rank p = 3.13e-14). Similar results showing that high-risk score patients had worse PFS (log-rank p = 6.95e-14) are shown in **Figures 4E, F**. The C-index for OS of the risk score model was 0.732 (95% CI: 0.684–0.779, p = 1.38e-21), and that for PFS was 0.728 (95% CI: 0.679–0.776, p = 3.11e-20). These results show that IPI-IPM has a good capacity for OS and PFS prediction. The distribution of the risk score, survival status, and the eight-gene expression between the high- and low-risk score groups is shown in **Supplementary Figures S3D, E** and **Figure 4G**. In addition, we conducted Spearman’s rank-order analysis to examine the correlation between IPI risk group and the eight-gene expression. As shown in **Supplementary Figure S3F**, the expression of almost all genes except PTX3 was significantly correlated with IPI risk groups. Then, we tested the difference of the eight-gene expression between high- and low-IPI risk groups. There existed significantly difference of gene expression in seven genes except PTX3.

**Table 2:** Univariate Cox regression analysis.

	Group	HR (95% CI)	p-value
Age Ref: >75	≤40	0.139 (0.041–0.469)	0.001
	41–60	0.372 (0.204–0.678)	0.001
	61–75	0.646 (0.369–1.130)	0.125
Ann Arbor clinical stage Ref: I–II	III–IV	1.760 (1.114–2.780)	0.015
ECOG performance status Ref: ≥2	0–1	0.395 (0.248–0.630)	<0.001
Gender Ref: Female	Male	1.149 (0.726–1.819)	0.554
LDH ratio Ref: >3	≤1	0.155 (0.072–0.333)	<0.001
	1–3	0.380 (0.185–0.781)	0.008
Number of extranodal sites Ref: ≥2	0–1	0.420 (0.243–0.727)	0.002
Risk level *^a^* Ref: High_Risk	Low_Risk	0.197 (0.120–0.324)	<0.001

HR, hazard ratio; 95% CI, 95% confidence interval.

a HR (95% CI) for continuous risk_score is 1.642 (1.464–1.842), p-value < 0.001.

**Figure 5:**
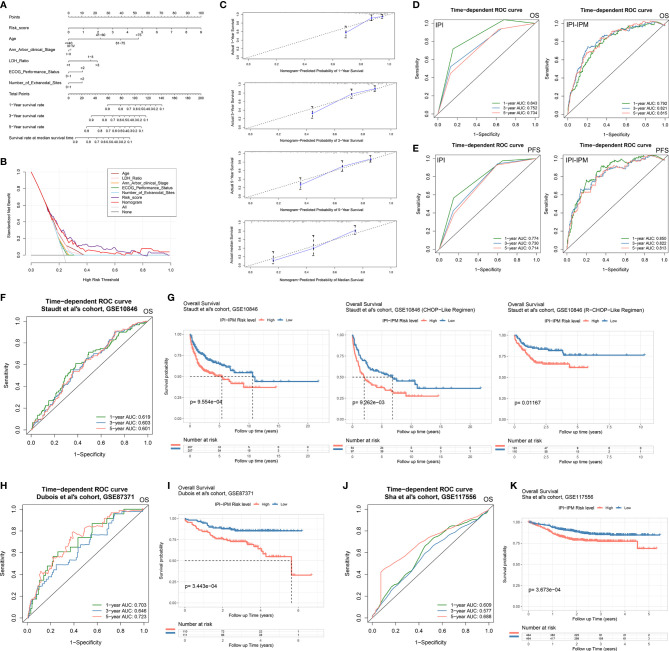
Construction of an IPI-based immune nomogram and validation of the IPI-IPM by using independent cohorts. **(A)** Nomogram for the prediction of the survival probability of 1-, 3-, and 5-year overall survival. **(B)** The DCA analysis of all parameters in the nomogram. **(C)** Calibration plots of nomogram-predicted probability of 1-, 3-, 5-year, and median survival. **(D)** Comparison of overall survival predictive ability between the IPI risk group and IPI-IPM *via* time-dependent ROC curve analysis. **(E)** Comparison of progression-free survival predictive ability between the IPI risk group and IPI-IPM *via* time-dependent ROC curve analysis. **(F–K)** Time-dependent ROC curve analysis of the IPI-IPM risk scores in validation cohorts on overall survival [**(F)** GSE10846, **(H)** GSE87371, **(J)** GSE117556] and Kaplan–Meier survival analysis of overall survival for patients of IPI-IPM high and low-risk groups in validation cohorts [**(G)** GSE10846, **(I)** GSE87371, **(K)** GSE117556].

Moreover, 335 patients with available clinicopathologic parameters were enrolled in the multivariate Cox regression analysis, presenting the risk scores and age as independent prognostic factors of OS (**Figure 4H**). Risk scores, along with age, Ann Arbor clinical stage, LDH ratio, ECOG performance status, and number of extranodal sites, were integrated to construct a nomogram model (**Figure 5A**). As shown in the decision curve analysis (DCA), the nomogram and the risk score from IPI-IPM showed a relatively high net benefit (**Figure 5B**). Moreover, the bias-corrected lines for the nomogram were close to the ideal line in the 1-, 3-, and 5-year and median survival time periods (**Figure 5C**). The C-index for the nomogram was 0.790 (95% CI: 0.736-0.843, p = 2.38e-26). Altogether, these results suggest that the nomogram has excellent capacity and consistency for OS prediction in the training cohort. Moreover, we collected patients with matched IPI-IPM risk score and available IPI information. A total of 445 patients with OS data and 386 patients with PFS data were enrolled to compare the difference of IPI and IPI-IPM in prognostic predictability. As shown in **Figures 5D, E**, the AUCs of time-dependent ROC for IPI-IPM were almost larger than those for the IPI risk group, indicating fairly equivalent prognostic predictability between IPI and IPI-IPM. In addition, the C-indices for OS of IPI-IPM and IPI were 0.749 (p = 3.78e-23) and 0.756 (p = 1.72e-13), and the C-indices for PFS were 0.739 (p = 6.36e-21) and 0.738 (p = 8.40e-11), respectively.

Three independent cohorts from different clinical centers (the cohort of Staudt et al., GSE10846, n = 414; the cohort of Dubois et al., GES87341, n = 221; the cohort of Sha et al., GSE117556, n = 928) were enrolled for further validation of IPI-IPM. The risk score for each patient was calculated, and all patients were divided into the high- and low-risk groups. As for the cohort of Staudt et al., the AUCs were 0.619, 0.603, and 0.601 for 1, 3, and 5 years, respectively (**Figure 5F**). Kaplan–Meier survival analysis showed significantly shorter OS of patients in the high-risk group (log-rank p = 5.30e-05, **Figure 5G** and **Supplementary Figures S3G, H**). Moreover, patients in the high-risk group were shown to have a shorter OS, regardless of the treatment regimens they received. Similar results of large AUC, shorter OS, and PFS of high-risk patients were also shown in the other two cohorts (**Figures 5H–K** and **Supplementary Figures S3F, G**). Taking the results of the training and testing cohorts together, the IPI-IPM and the nomogram combined risk score with relevant clinical characteristics (age, Ann Arbor clinical stage, LDH ratio, ECOG performance status, and number of extranodal sites) was an excellent model for predicting short-term or long-term OS in DLBCL patients, which may guide therapeutic strategy decisions and long-term prognosis.

## Molecular Characteristics of IPI-IPM Subgroups

**Figure 6:**
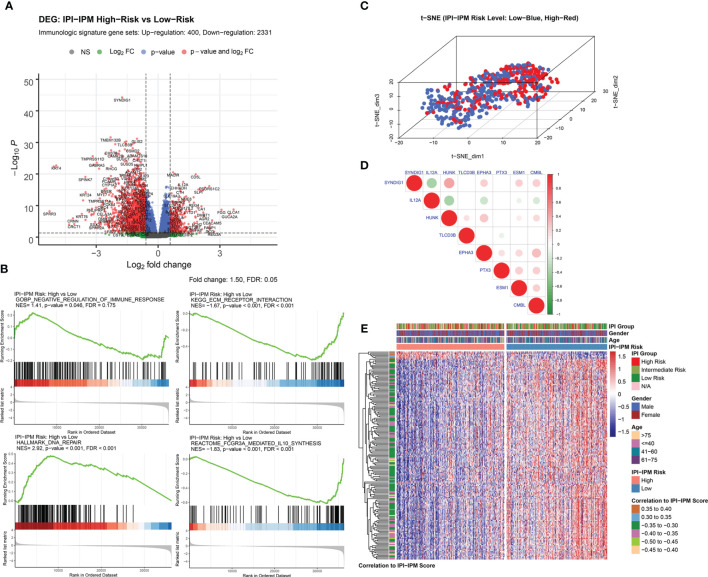
Gene expression analysis of IPI-IPM and identification of IPI-IPM-associated immune genes. **(A)** Volcano plot of immune-related DEGs between the high and low IPI-IPM risk groups. **(B)** Pre-ranked GSEA of enriched gene sets between the high and low IPI-IPM risk groups. **(C)** The t-SNE algorithm was applied to show the gene expression diversity between DLBCL patients in the high and low IPI-IPM risk groups. **(D)** Pearson correlation analysis of the eight genes composing the IPI-IPM. **(E)** Heatmap of immune-related genes of which expression correlate with the IPI-IPM risk score.

Compared to the low-risk group, a total of 5,980 genes (690 upregulated and 5,290 downregulated), and 2,731 immunologic genes (400 upregulated and 2,331 downregulated) were significantly differentially expressed in the high-risk group (**Supplementary Figure S4A**, **Figure 6A**, and **Supplementary Material 6**). Pre-ranked gene set enrichment analysis (GSEA) was performed to show that several gene sets, including negative regulation of immune response, DNA repair, and response to IL-12, were enriched in the high-risk group, and several gene sets, including ECM receptor interaction, IL-10 synthesis, and regulation of humoral immune response, were enriched in the low-risk group. Details are documented in **Figure 6B**, **Supplementary Figures S4B–E** and **Supplementary Material 6**. Additionally, t-distributed stochastic neighbor embedding (t-SNE) was applied to show obvious genetic diversity between the high- and low-risk groups (**Figure 6C**). Based on the Pearson correlation analysis, there was either a positive or negative correlation between the eight genes from the IPI-IPM (**Figure 6D**). **Figure 6E** presents the heatmap of differentially expressed genes correlating IPI-IPM risk scores between the high- and low-risk groups.

**Figure 7:**
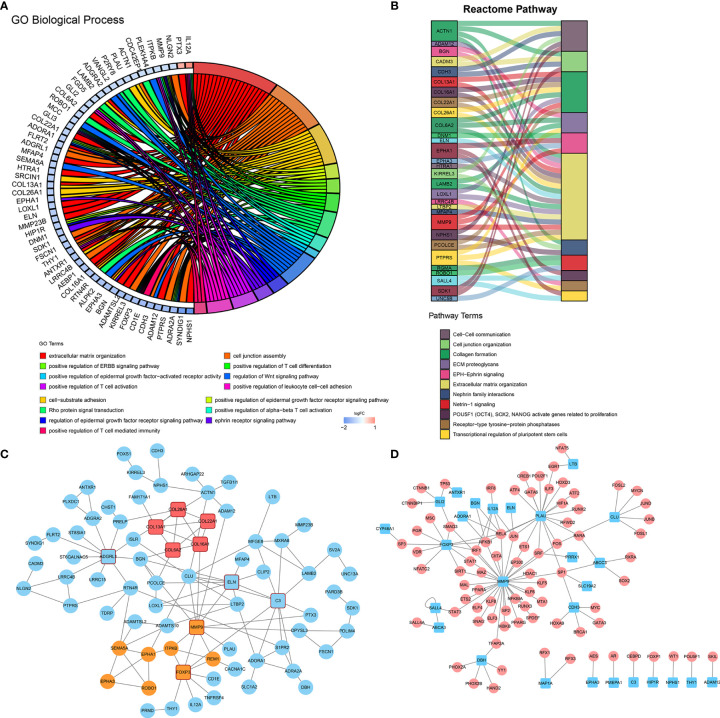
Molecular characteristics of IPI-IPM-associated immune genes. **(A, B)** Over-representative analysis: a chord map of the enriched GO biological processes and a Sankey plot of the enriched Reactome pathway terms. **(C)** Protein–protein interaction network (PPI) analysis based on the STRING database. **(D)** Transcription factor network analysis based on the TFTRUST database.

To gain further molecular insight into the molecular characteristics of IPI-IPM, 176 genes correlating with risk scores and the eight genes from the IPI-IPM were identified as IPI-IPM-associated immune genes (absolute Pearson correlation coefficient ≥ 0.3, FDR < 0.05). Overrepresentation analysis was applied to identify the enriched biological functions and pathways, such as ECM organization and T cell differentiation, activation, and mediated immunity (**Figures 7A, B**). Detailed results are listed in **Supplementary Material 6**. As shown by the PPI network, the ECM organization, oncogenesis, and tumor immunity-associated regulatory genes (*COL6A2*, *COL16A1*, *COL26A1*, *COL13A1*, *COL22A1*, *C3*, *ELN*, *MMP9*, *CLU*, *FOXP3*, and *ADGRL1*) were closely correlated, and acted as hub genes (**Figure 7C**). In addition, the TFTRUST database was used to explore the transcription factors (TFs) regulating the 184 IPI-IPM-associated immune genes; thus, *MMP9*, *FOXP3*, and *PLAU* were identified in the TF network (**Figure 7D**).

**Figure 8:**
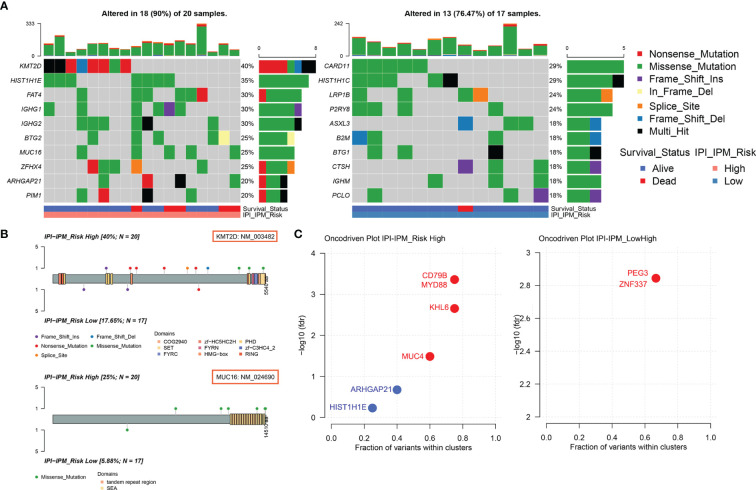
Somatic mutational analysis of the high and low risk IPI-IPM groups. **(A)** Mutated genes of high and low IPI-IPM risk groups. Top 10 mutated genes (rows) are ordered by mutation rate. The color-coding legends indicate the mutation types and survival status of patients. **(B)** Lollipop plots for amino acid changes of KMT2D and MUC16. **(C)** Oncodrive plots of the high and low risk IPI-IPM groups.

Based on the somatic mutational data of 37 samples from TCGA, 18 out of 20 patients in the high-risk group and 13 out of 17 patients in the low-risk group were found to have altered gene expression (**Supplementary Figures S5A, B** and **Figure 8A**). Although most mutations were missense mutations, more nonsense mutations and other mutations were identified in the high-risk group (**Figure 8A**). In addition, the mutation frequency of the top 10 genes in the high-risk group was much higher than that in the low-risk group. Furthermore, we investigated specific mutation sites of key genes corresponding to their amino acid location, including *KMT2D*, *MUC16*, *CARD11*, *LRP1B*, *BTG2*, and *PIM1* (**Figure 8B** and **Supplementary Figure S5C**). As shown in the Oncodrive plot, *MYD88*, *CD79B*, *KHL6*, and *MUC4* were identified as cancer driver genes in the high-risk group whereas only *PEG3* and *ZNF337* were identified in the low-risk group (**Figure 8C** and **Supplementary Material 7**).

### Immune Landscape of IPI-IPM Subgroups

**Figure 9:**
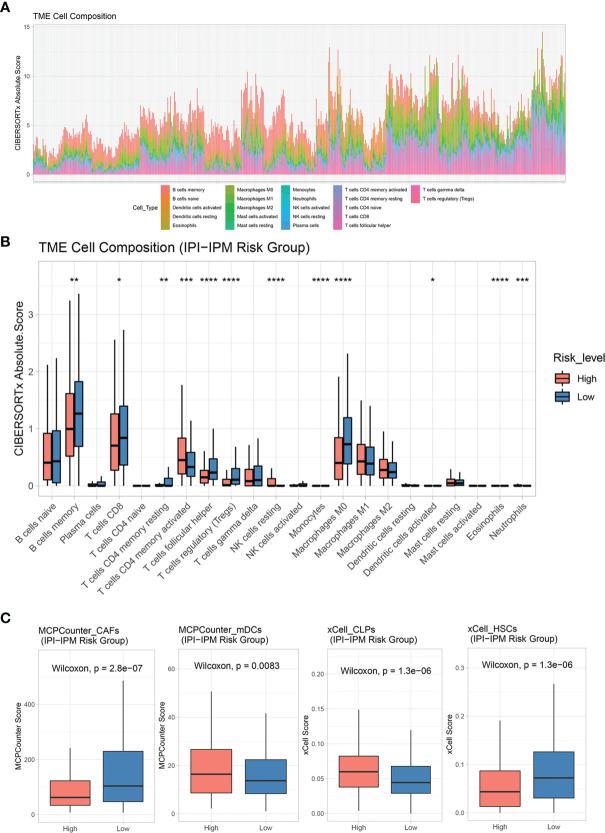
Tumor immune microenvironment characteristics of IPI-IPM subgroups. **(A, B)** Analysis of immune cell infiltration by using the CIBERSORTx algorithm: relative proportion of each type of cell infiltration in DLBCL patients and bar plots for visualization of significantly differentially TME-infiltrating cells between high and low IPI-IPM risk groups. **(C)** Analysis of immune cell infiltration by using the MCPcounter and xCell algorithm. P value, * < 0.05, ** < 0.01, *** < 0.001, **** < 0.0001.

CIBERSORT was applied to analyze the infiltrating abundance of various immune cell types in the different IPI-IPM subgroups (**Figure 9A** and **Supplementary Figure S6A**). Activated memory CD4^+^ T cells and resting NK cells showed high infiltration in the high-risk group, whereas memory B cells, CD8^+^ T cells, follicular helper T cells, regulatory T cells (Tregs), and non-activated macrophages were more abundant in the low-risk group (**Figure 9B**). In addition, the MCPCounter and xCell algorithms were applied to show that myeloid dendritic cells (mDCs) and common lymphoid progenitors showed high infiltration in the high-risk group, whereas hematopoietic stem cells, cancer-associated fibroblasts (CAFs), and T cells, especially CD8^+^ T cells, were more abundant in the low-risk group (**Figure 9C** and **Supplementary Figure S6B**).

**Figure 10:**
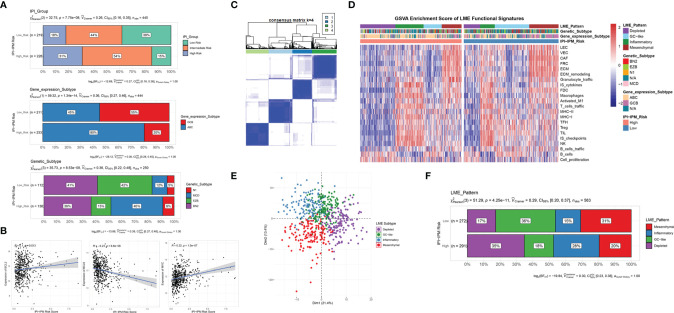
Molecular and TME subtypes for DLBCL of IPI-IPM subgroups. **(A)** Distribution of IPI groups, gene expression subtypes, and genetic subtypes between high and low IPI-IPM risk groups. **(B)** Correlation analysis of the IPI-IPM risk score and Bcl-2, Bcl-6, and c-Myc. **(C)** Consensus clustering to detect lymphoma microenvironment (LME) clusters. **(D, E)** Distribution of LME patterns between high and low IPI-IPM risk groups. **(F)** GSVA enrichment score of LME functional signature between high and low IPI-IPM risk groups.

In addition, DLBCL subtypes varied between the high- and low-risk groups. As shown in **Figure 10A**, more patients with higher IPI levels were classified into the IPI-IPM high-risk group, with similar results for age, Ann Arbor clinical stage, LDH ratio, ECOG performance status, and the number of extranodal sites (**Supplementary Figure S6C**). The high-risk group contained a higher proportion of activated B-cell-like DLBCLs, whereas the low-risk group was more enriched in germinal center B-cell-like DLBCLs (p = 1.34e-14). Schmitz et al. (4) identified four prominent genetic subtypes in DLBCL with different responses to immunochemotherapy: MCD (the co-occurrence of MYD88^L265P^ and CD79B mutations), BN2 (BCL6 fusions and NOTCH2 mutations), N1 (NOTCH1 mutations), and EZB (EZH2 mutations and BCL2 translocations), and uneven distributions of these four subtypes were found between the high- and low-risk groups. For example, the poor-prognostic MCD subtype represented 40% of the high-risk group and 12% of the low-risk group. In addition, the correlation analysis showed that IPI-IPM risk scores were positively correlated with the expression of *BCL-2* and *MYC* and negatively correlated with *BCL-6* expression (**Figure 10B**). Taken together, these data indicate that IPI-IPM provides additional orthogonal information from previous lymphoma classifications.

To further explore the interaction between DLBCL cells and the microenvironment, Kotlov et al. (11) defined the LME into four major transcriptionally defined categories with distinct biological properties and clinical behavior, including GC, MS, IN, and DP forms. Similarly, we utilized an unsupervised clustering method to assign the samples into four groups by using expression data from the 22 functional gene expression signature (F^GES^) sets (**Figure 10C**). GSVA enrichment scores were calculated to demonstrate distinct TME characteristics among the four biological patterns (**Figure 10D**). Consistent with the CIBERSORT results, the DP LME categories represented 35% of the high-risk group whereas the GC-like and MS LME categories were more enriched in the low-risk group (**Figures 10E, F** and **Supplementary Material 8**).

### Potential Therapeutic Value of IPI-IPM

**Figure 11:**
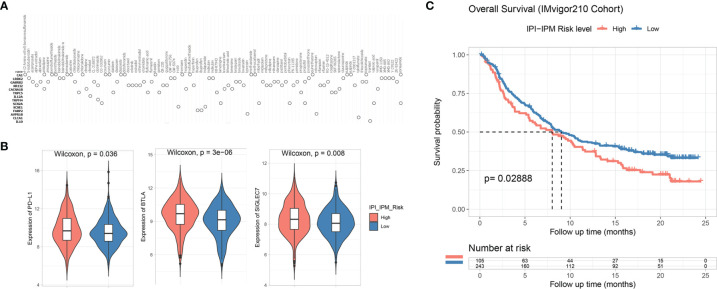
Potential therapeutic value based on IPI-IPM. **(A)** Connectivity map (CMap) results of top IPI-IPM associated immune genes. **(B)** The expression of inhibitory immune checkpoints between high and low IPI-IPM risk groups. **(C)** Kaplan–Meier survival analysis of overall survival for patients of IPI-IPM high and low risk groups in the IMvigor210 Cohort.

To further understand the effects of the risk score on drug response, 184 IPI-IPM-associated immune genes were mapped into the connectivity map database (17). As shown in **Figure 11A**, 14 genes (*ADORA1*, *ADRA2A*, *CACNA1C*, *DBH*, *DNM1*, *ELN*, *ENPP1*, *EPHA1*, *IL12A*, *MMP9*, *PLAU*, *S1PR2*, *SLC1A2*, and *SV2A*) were associated with 122 inhibitors, involving 54 mechanisms of action. The details are documented in **Supplementary Material 9**.

The clinical development of cancer immunotherapy and the advances in genomic analysis have validated the important role of the TME in predicting cancer response to ICB therapy (13, 18, 19). We investigated the expression of several inhibitory immune checkpoints between the high- and low-risk groups by using the normalized gene expression data (VST transformed). As shown in **Figure 11B**, the expression of *PD-L1*, *BTLA*, and *SIGLEC7* was significantly upregulated in the high-risk group. Expressions of other inhibitory immune checkpoints such as B7-H3 were found downregulated in the high-risk group (**Supplementary Figure S6D**).

Various biomarkers were reported to predict the response to immunotherapy, including tumor mutation burden and expression of immune checkpoints, such as PD-L1. We examined the value of the IPI-IPM to predict the response of patients to ICB therapy based on a publicly accessible dataset, the IMvigor210 Cohort (20). Using an optimal cutoff point calculated through the “surv_cutpoint” function (a minimal proportion of observations per group was set to 30%) of the survminer R package for group assignment, we observed that patients with high IPI-IPM risk scores had shorter OS after anti-PD-L1 treatment (log-rank p = 0.029, **Figure 11C**).

## Discussion

Recent groundbreaking insights into the pronounced genomic heterogeneity of DLBCL have identified potential biomarkers for patient diagnosis and prognosis, paving the way for a standardized application of precise medicine (3, 5, 13). Multiple subtype classifications as well as IPI and enhanced NCCN-IPI were built to stratify prognostically relevant subgroups of DLBCL patients with R-CHOP therapy, whose robustness requires further investigation in the context of targeted therapies (7, 11, 13, 21). In the current study, we used WGCNA to profile IPI correlating immune gene sets and constructed and validated an eight-gene IPI-IPM (*CMBL*, *TLCD3B*, *SYNDIG1*, *ESM1*, *EPHA3*, *HUNK*, *PTX3*, and *IL12A*) with shorter OS in the high-risk patients and longer OS in the low-risk patients in both TCGA and three independent cohorts.

*ESM1*, also known as endocan, has been shown to regulate endothelial cell function in the initiation and progression of human cancers, including esophageal cancer, hepatocellular carcinoma, bladder cancer, and breast cancer (22). The a*EphA3* receptor plays a critical role in cell adhesion and migration during development and homeostasis of many tissues as well as in cancer growth, progression, and angiogenesis (23). In addition, *EphA3* is highly expressed in tumors, but not in normal tissues, which, together with antitumor properties of anti-EphA3mAb (chIIIA4), defined *EphA3* as a potential target for antibody-based anticancer therapies (23). *PTX3* is secreted by dendritic cells, macrophages, and fibroblasts and is actively involved in the regulation of inflammation, tissue remodeling, and cancer (24). *PTX3* interacts with the PI3K/AKT/mTOR signaling pathway or the fibroblast growth factor-2/receptor system to regulate tumor cell proliferation, apoptosis, and metastasis in lung cancer, breast cancer, melanoma, prostate cancer, and multiple myeloma (24, 25). *IL12A* is a potent immunosuppressive cytokine produced by regulatory B cells, Treg cells, macrophages, dendritic cells, and tumor cells, which suppresses the effector functions of CD4^+^ and CD8^+^ T cells but strongly favors Treg proliferation (26). Furthermore, Larousserie et al. found that high levels of IL12A were associated with poor survival in DLBCL patients (27).

High-throughput gene expression databases and bioinformatics analysis have enabled systematic profiling of prognostic signatures in DLBCL. For example, Zhou et al. uncovered differentiated lncRNA expression patterns between germinal center B-cell-like and activated B-cell-like DLBCL and identified an immune-associated 17-lncRNA signature for subtype classification and prognosis prediction (28). Chapuy et al. defined five distinct DLBCL subsets by integrating recurrent mutations, somatic copy number alterations, and structural variants (5). Hu et al. built a predictive model combining drug resistance signature with clinical factors, including age at diagnosis, stage, number of extra nodal sites, and ECOG performance score (21). In the current study, IPI-IPM risk score remained an independent prognostic factor after modification of clinical characteristics; thus, we developed a nomogram model combining the risk score and other clinical features (age, Ann Arbor clinical stage, LDH ratio, ECOG performance, and number of extranodal sites) to predict the OS probability of DLBCL patients in 1, 3, and 5 years, and the median survival time. Both the calibration curve and DCA analysis supported the notion that our nomogram provides a complementary perspective on individualizing tumors and develops an individual scoring system for patients, thus making it a promising tool for clinicians in the future.

In the GSEA analysis of the high- and low-risk groups, several immune-related gene sets, including negative regulation of immune response, B cell and T cell proliferation, response to IL-12 and γ-IFN, and NOD-like and TOLL-like receptor signaling, were enriched in the high-risk group whereas ECM receptor interaction, IL-10 synthesis, and regulation of humoral immune response were enriched in the low-risk group. Therefore, we speculated that the local immune signature conferred a weaker immune phenotype in the high-risk group, but an intense immune phenotype in the low-risk group. Moreover, overrepresentation analysis identified several immune-related pathways, including ECM organization and T cell activation, which were enriched with IPI-IPM-associated immune genes. The ECM plays important roles in supporting cells and regulating intercellular interactions, thus contributing to the progression of several malignancies (11, 14). Lenz et al. built a survival model with two stromal gene signatures for DLBCL patients who received CHOP or R-CHOP, where the prognostically favorable stromal-1 signature reflected ECM deposition and histiocytic infiltration, whereas the prognostically unfavorable stromal-2 signature reflected tumor blood vessel density (12).

To further explore the immunological nature of the IPI-IPM subgroups, we analyzed the somatic mutational profiles of 37 samples and found higher mutation counts in the high-risk group with more nonsense mutations, although missense mutations were the most common type. The largest difference in mutations between high- and low-risk groups was in the *KMT2D* mutation (40% in high-risk samples *vs*. 17.65% in low-risk samples) (29). *KMT2D* is a tumor-suppressor gene in DLBCL, and genetic ablation of *KMT2D* in a *BCL2*-overexpression-driven model promotes higher DLBCL penetrance (30). Moreover, *MYD88* and *CD79B* were identified as cancer driver genes in the high-risk group, which is in good agreement with the previous findings that *MYD88* and *CD79B* mutations have been associated with tumor response and survival in DLBCL patients (31–33). Ngo et al. initially identified activating *MYD88* mutations in DLBCL, where L265P was the most frequent and oncogenic form. *MYD88* was shown to interact with *IRAK1* and *IRAK4* and activate the NF-κB and JAK-STAT3 pathways, promoting malignant cell proliferation and causing worse survival of DLBCL patients (34). *CD79B* mutations are frequently detected in the first tyrosine (Y196) of the immunoreceptor tyrosine-based activation motif. *CD79B* mutations were shown to cause chronic activation of BCR signaling and constitutive NF-κB activation, further promoting tumor cell growth within the immunosuppressive TME.

Consistently, the composition of immune cells was different between the two IPI-IPM subgroups, where memory B cells, CD8^+^ T cells, Tregs, non-activated macrophages, and CAFs were more abundant in the low-risk group. It is generally accepted that cytotoxic CD8^+^ T cells, following successful priming, recognize tumor-specific (neoantigens) or tumor-associated antigens and exert antitumor function primarily *via* the release of cytotoxic molecules such as perforin and granzymes (35–37). However, Tregs suppress CD8^+^ T cells by direct cell contact and secretion of inhibitory cytokines including IL-10 and TGF-β (36, 38). TAMs have been shown to mediate antibody-dependent cellular phagocytosis of rituximab in malignant B cells, limit CD8^+^ T cell activity through PD-L1 expression, and release IL-10 and TGF-β or inhibiting enzymes, thus regulating antitumor immunity and response to therapy (39). CAFs, the resident fibroblasts activated in a chronically inflamed TME, have been shown to promote recruitment and polarization of regulatory cells including Tregs, monocytes, and M2-macrophages by secreting IL-6, CXCL12, Chi3L1, MCP-1, and SDF-1, thus actively shaping immune infiltration in the TME (40). In addition, CAFs have been shown to impact the cytolytic activity of CD8^+^ T cells through different mechanisms, such as the production of prostaglandin E2 and nitric oxide to dampen CD8^+^ T cell proliferation, expression of PD-L2 and FasL to promote CD8^+^ T cell apoptosis, and induction of abnormal ECM deposition and remodeling to physically trap CD8^+^ T cells and prevent effective tumor access (41).

In addition, DP LME represented more of the high-risk group, whereas immune-rich GC LME was more enriched in the low-risk group. Kotlov et al. characterized the DLBCL microenvironment by analyzing gene expression profiles, developing TME-derived F^GES^, analyzing ECM composition by proteomics, and establishing patient-derived tumor xenograft models (11). Four basic categories of DLBCL LME with distinct clinical and biological connotations were identified to uncover the bidirectional interaction between DLBCL cells and the LME. Remarkably, immune-rich GC LME confers a better prognosis than DP LME, suggesting the fundamental role of LME in preventing lymphomagenesis. In turn, DLBCL cells develop genetic and epigenetic traits that contribute to immune evasion from LME.

Finally, we applied Spearman correlation analysis to estimate the potential therapeutic effects of IPI-IPM and explored the expression of several inhibitory immune checkpoints between IPI-IPM subgroups to predict the response to immunotherapy. The results showed that IPI-IPM-associated genes were correlated with sensitivity to drugs targeting the Aurora kinase, DNA methyltransferase, histone acetyltransferase, FLT3, EGFR, and VEGFR signaling pathways, indicating that high-risk DLBCL patients may benefit from novel inhibitors targeting these signaling pathways. As for the expression of inhibitory checkpoints, *PD-L1*, *BTLA*, and *SIGLEC7* were significantly upregulated in the high-risk group. Upregulation of checkpoints (*PD-1*, *CTLA-4*, and *TIM-3*) and their ligands (PD-L1 and PD-L2) in the TME can mediate tumor cells to escape immune surveillance by modulating T-cell activity (13, 18, 42, 43). Thus, ICB exerted significant antitumor effects in both solid tumors and hematologic malignancies. Although nivolumab monotherapy showed low efficacy in unselected DLBCL patients, pembrolizumab combined with R-CHOP was safe and associated with a high complete response rate and improved 2-year progression-free survival (19, 44). *BTLA* has been reported to mark a high-checkpoint-expressing T-cell subset (*PD-1*, *TIM-3*, *LIGHT*, and *LAG-3*) with decreased cytolytic function and increased proliferation ability, thus correlating with poor prognosis in DLBCL (13, 45).

Taken together, the IPI-IPM risk score was compatible with the ability of tumor-infiltrating immune cells to determine the expression of immune checkpoints, suggesting that the poor prognosis of the high-risk group may be due to the stronger immunosuppressive TME and that high-risk patients will benefit more from immune checkpoint inhibitors than low-risk patients, resulting in a better prognosis. Our research provides new insights into the TME and immune-related therapies for DLBCL. However, it is noteworthy that some limitations arose because the conclusions were drawn from data from retrospective studies, and prospective studies are warranted to further confirm our results. In addition, functional and mechanistic studies should be conducted to support their clinical application of the genes in our risk model. 

## Materials and Methods

### Data Selection and Acquisition

Data acquisition of the present study is fully under the TCGA publication guidelines (https://www.cancer.gov/tcga). Gene expression data (RNA-seq) of 570 samples, masked somatic mutation data of 37 samples, and clinical follow-up data with clinicopathological characteristics of 566 patients of DLBCL projects (TCGA-DLBC, CTSP-DLBCL1, NCICCR-DLBCL) were obtained from the Cancer Genome Atlas (TCGA) by using the TCGAbiolinks (46) R package in R software (version 4.0.2, https://www.r-project.org). Clinical information, gene expression subtype, and genetic subtype of the DLBCL patients were supplemented by referring to open-access supplementary files of the GDC DLBCL publication (4). Matched gene expression data and survival follow-up data can be obtained in 563 samples. Matched gene expression data and survival follow-up data with available IPI data can be obtained in 445 samples (**Supplementary Table 1**). Gene expression data (Microarray) with matched clinical information of 414, 221, and 928 DLBCL patients were obtained from GSE10846, GSE87371, and GSE117556, respectively (12, 47–49), by accessing the Gene Expression Omnibus (GEO) database (www.ncbi.nlm.nih.gov/geo). The immunologic gene lists used in the present study were downloaded from the ImmPort (www.immport.org/shared/home) and ImmuneSigDB (50) through MSigDB Collections (www.gsea-msigdb.org/gsea/msigdb). For the RNA-Seq data, HTSeq-count data were downloaded. The Combat-Seq function of the Sva (51) R package was used to remove batch effects among different projects. The principal component analysis was performed and visualized to examine the batch effect (52). For each gene, the effective gene length was extracted by using the EDASeq (53) R package and TPM (Transcripts Per Kilobase Million) gene expression data were calculated through the count2TPM function utilized in the IOBR (54) R package by using the corresponding effective gene length. A Homo sapiens GRCh38 annotation file (Ensembl 103) downloaded from Ensembl (55) was used for gene symbol annotation. The DESeq2 (56) R package was applied for the normalization of RNA-seq count data, and variance stabilizing transformation (VST) data were used for downstream analysis. DLBCL microenvironmental (LME) signatures and LME subtype categorizing method were referred to the publication of Kotlov et al. on Cancer Discovery (11).

### Identification of Differentially Expressed Genes

Samples with available IPI information were categorized to high-, intermediate-, and low-risk groups according to criteria of IPI, and the DESeq2 (56) R package was applied to identify differentially expressed genes (DEGs) between high- and low-risk groups. The differential expression was defined with a fold change of threshold at 1.5 and a false discovery rate (FDR) value < 0.05.

### Gene Functional Enrichment Analysis

The clusterProfiler (57) R package was used for both overrepresentation analysis and pre-ranked gene set enrichment analysis (GSEA). Analysis of Gene Ontology (GO) (58), Kyoto Encyclopedia of Genes and Genomes (KEGG) pathway (59), and Reactome pathway (60) were contained in the present study. An adjusted p value<0.05 was considered statistically significance. The threshold for GSEA was set at the p-value < 0.05, FDR < 0.05, and | normalized enrichment score (NES) | > 1.0. The non-parametric gene set variation analysis was further performed with the GSVA (61) package of R.

### Weighted Gene Co-Expression Network Analysis

Weighted gene co-expression network analysis (WGCNA) is commonly used for analyzing high-throughput gene expression data with different characteristics, so as to mine gene co-expression networks and intramodular hub genes based on pairwise correlations in genomic applications. In the present study, we applied the WGCNA (62, 63) R package to analyze key gene clusters that were most relevant to IPI risk groups in DLBCL samples. 

### Construction and Validation of IPI-Based Immune-Related Prognostic Model

IPI-based immune-related genes were selected to construct the prognostic risk model. The training cohort (563 patients with matched normalized RNA-seq data and survival data from TCGA) was used for the construction of IPI-IPM, and three testing cohorts (GSE10846, GES87341, and GSE117556) were used for validation of the prognostic risk model. The Survival R package was used to analyze the correlation between the expression of objective gene sets and DLBCL patients’ overall survival (OS). Univariate Cox regression analysis was performed to screen genes, of which the expression was associated with OS with a p value < 0.05. Lasso (least absolute shrinkage and selection operator) regression analysis was applied for variable selection and regularization to enhance the prediction accuracy and interpretability by using the glmnet (64) R package. Multivariate Cox regression analysis was then carried out to select the optimal genes, based on the method of the Akaike information criterion (AIC) (65). For each sample, the risk score equals the sum of the normalized expression of each gene multiplying its corresponding regression coefficient. Time-dependent ROC curves were plotted by using the survivalROC (66) R package. Five hundred sixty-three DLBCL patients in the training cohort were divided to low- and high-risk score groups according to the optimal cutoff value with largest AUC in the receiver operating characteristic (ROC) curve of the median survival time. Then, Kaplan–Meier survival analysis and time-dependent ROC curve analysis were performed to evaluate the prognostic significance and accuracy of IPI-IPM (67). Besides, Harrell’s concordance index (C-index) was calculated by using the survcomp (68) R package. Univariate and multivariate Cox regression analyses were performed on the risk score and all available clinicopathologic parameters, including age, gender, Ann Arbor clinical stage, LDH ratio, ECOG performance status, and number of extranodal site. Then, we utilized the rms (69) R packages which set up a prognostic nomogram for OS probability assessment by enrolling all the independent prognostic factors. The discriminative efficacy, consistency, and clinical judgment utility of the nomogram score was evaluated by time-dependent calibration plots and decision curve analysis (DCA) (70) using the rms and rmda (71) R package.

### Comprehensive Analysis of Molecular and Immune Characteristics in Different IPI-IPM Subgroups

DEGs between high- and low-risk score groups were analyzed following the fold change of a threshold at 1.5 and FDR value < 0.05. The gene expression of samples between IPI-IPM subgroups were analyzed with the t-distributed stochastic neighbor embedding (t-SNE) method by using the Rtsne (72) package of R and then visualized on the 3D map with the scatterplot3d (73) package of R. Somatic mutations of IPI-IPM subgroups were analyzed by using the Maftools (74) R package. The intersection of DEGs and immune-related gene set was used to construct a protein–protein interaction (PPI) network based on the STRING (75) database. Cytoscape (76), plugin MCODE (77), and cytoHubba (78) were utilized to identify top 10 degree genes in the network. The TRRUST (79) database was browsed to explore the curated transcriptional regulatory networks. Gene expression data (TPM) of 570 DLBCL samples were imported into CIBERSORT (80), MCPCounter (81), and xCell (82) to calculate the score to estimate the proportion of TME cells including immune and stromal cells. 

### Clustering Analysis of Expression Pattern of LME Signatures

An unsupervised clustering algorithm was applied to analyze the gene expression pattern of LME signatures in 563 DLBCL samples. By using the ConsensusClusterplus (83) R package, we performed the k-means clustering algorithm with 1,000 repetitions to ensure the stability. The Pearson distance matrix calculated from the clustering was then imported to Cytoscape for the visualization of distribution of samples corresponding to LME signature clustering. 

### Data Analysis

All statistical data were analyzed in the R software (version 4.0.3). A Wilcoxon test was applied to compare continuous variables between two groups of sample data. A Kruskal–Wallis test was applied to compare continuous variables among three or more groups of sample data. A test is considered with statistical significance at two-sided p < 0.05. Pearson correlation was used to test the correlation between two sets of continuous data, and an absolute Pearson correlation coefficient larger than 0.3 was considered to be correlated. A Pearson correlation is considered with statistical significance at FDR < 0.05. Spearman correlation was used to test the correlation between gene expression and IPI groups. We used ggplot2, ggstatsplot, and ggpubr R packages (84, 85) for data analysis and visualization. 

## Data Availability Statement

The datasets presented in this study can be found in online repositories. The names of the repository/repositories and accession number(s) can be found in the article/**Supplementary Material**.

## Ethics Statement

The patient cohorts we used were publicly available datasets that were collected with patients’ informed consent.

## Author Contributions

SM, DS, CS, and YH conceived of and designed the study. SM and DS did the literature research, performed the study selection, data extraction, and statistical analysis, and wrote the draft. LA, FF, and FP participated in the extraction and analysis of data. All authors contributed to the article and approved the submitted version.

## Funding

This work was supported by grants from the National Natural Science Foundation of China (No. 81974007 to CS) and the National Key R&D Program of China (Grant No. 2019YFC1316204 to YH).

## Conflict of Interest

The authors declare that the research was conducted in the absence of any commercial or financial relationships that could be construed as a potential conflict of interest.

## Publisher’s Note

All claims expressed in this article are solely those of the authors and do not necessarily represent those of their affiliated organizations, or those of the publisher, the editors and the reviewers. Any product that may be evaluated in this article, or claim that may be made by its manufacturer, is not guaranteed or endorsed by the publisher.

## Supplementary Material

Supplementary Figure 1 Identification of DEGs of different IPI risk groups and gene functional enrichment analysis. **(A)** Volcano plot of differentially expressed genes (DEGs) between the high and low IPI Risk groups. **(B)** Heatmap of differentially expressed genes (DEGs) between the high and low IPI Risk groups. **(C)** Venn plot of intersection of DEGs and immune-related gene sets. **(D)** Heatmap of differentially expressed immune-related genes (DEGs) between the high and low IPI Risk groups. **(E)** Over representative analysis of the differentially expressed immune-related genes on GO biological process, KEGG and Reactome Pathway terms. **(F)** Comparation of GSVA results of IPI Risk groups on immune-related pathways.

Supplementary Figure 2 WGCNA for the identification of modules related to IPI risk group and gene functional enrichment analysis for module genes. **(A–D)** Process of sample clustering and outlier detection, sample dendrogram, calculation of Beta value and soft thresholding, and clustering of module eigengenes. **(E)** Correlation between gene module membership and gene significance for IPI risk group in Darkgreen, Blue and Lightyellow modules. **(F, G)** Heatmaps of the Topological Overlap Matrix (TOM) and Eigengene adjacency. **(H)** Venn plot of intersection of IPI related module genes identified by WGCNA and immune-related gene sets. **(I)** Over representative analysis of the intersected module genes on GO biological process, KEGG and Reactome Pathway terms.

Supplementary Figure 3 Construction and validation of an IPI-based immune prognostic model. **(A)** Plot for the estimated coefficients with respect to the change of the penalty parameter of the Lasso penalized Cox regression. **(B)** Kaplan-Meier Survival analysis of OS and PFS for patients of high and low IPI-IPM risk groups (median as cutoff point). **(C)** Plot for median survival time of training cohort. **(D** and **E)** Risk scores and survival status of patients in high and low IPI-IPM risk groups. **(F** and **G)** Time-dependent ROC curve analysis of the IPI-IPM risk scores and Kaplan-Meier Survival analysis for patients of IPI-IPM high and low risk groups on PFS in validation cohorts (**F**: GSE87371 and **G** GSE117556). **(H)** Spearman correlation analysis of gene expression and IPI Risk group and difference test of gene expression between high and low IPI Risk group for the eight genes.

Supplementary Figure 4 Gene expression analysis of IPI-IPM and gene functional enrichment analysis. **(A)** Volcano plot of DEGs between high and low IPI-IPM risk group. **(B–E)** Pre-ranked GSEA of enriched gene sets between high and low IPI-IPM risk group on GO biological process, Hallmark gene sets, KEGG and Reactome Pathway terms.

Supplementary Figure 5 Somatic mutational profiles of IPI-IPM subgroups. **(A** and **B)** SNV landscape and top mutated genes of all included samples. **(B)** Lollipop plots for amino acid changes of selected genes.

Supplementary Figure 6 TME characteristics, clinicopathological features and expression of inhibitory immune checkpoints in IPI-IPM subgroups. **(A)** Composition of immune cells infiltration by using the CIBERSORT algorithm. **(B)** Analysis of specific types of infiltrating immune cells by using the MCPcounter and xCell algorithm. **(C)** Relation of IPI-IPM Risk group and DLBCL clinicopathological features. **(D)** The expression of multiple inhibitory immune checkpoints between high and low IPI-IPM risk groups.

Supplementary Table 1 Data Source and Clinical Table.

Supplementary Table 2 Immunologic signature gene sets.

Supplementary Table 3 DEG of IPI High and Low Risk group and over-representative analysis.

Supplementary Table 4 WGCNA results and over-representative analysis.

Supplementary Table 5 Construction of IPI-IPM.

Supplementary Table 6 Analysis Results of IPI-IPM (DEG, GSEA, ORA and PPI).

Supplementary Table 7 Onco-driven mutation and TMB.

Supplementary Table 8 TME pattern analysis and DLBC subtypes data.

Supplementary Table 9 Connectivity map results of top enriched immune related genes.

## References

[1] Wang L Li LR Young KH. New Agents and Regimens for Diffuse Large B Cell Lymphoma. J Hematol Oncol (2020) 13:175. doi: 10.1186/s13045-020-01011-z 33317571 PMC7734862

[2] Solimando AG Annese T Tamma R Ingravallo G Maiorano E Vacca A. New Insights Into Diffuse Large B-Cell Lymphoma Pathobiology. Cancers (Basel) (2020) 12(7):1869. doi: 10.3390/cancers12071869 PMC7408689 32664527

[3] El Hussein S Shaw KRM Vega F. Evolving Insights Into the Genomic Complexity and Immune Landscape of Diffuse Large B-Cell Lymphoma: Opportunities for Novel Biomarkers. Mod Pathol (2020) 33:2422–36. doi: 10.1038/s41379-020-0616-y 32620919

[4] Schmitz R Wright GW Huang DW Johnson CA Phelan JD Wang JQ. Genetics and Pathogenesis of Diffuse Large B-Cell Lymphoma. New Engl J Med (2018) 378:1396–407. doi: 10.1056/NEJMoa1801445 PMC6010183 29641966

[5] Chapuy B Stewart C Dunford AJ Kim J Kamburov A Redd RA. Molecular Subtypes of Diffuse Large B Cell Lymphoma Are Associated With Distinct Pathogenic Mechanisms and Outcomes. Nat Med (2018) 24:679–90. doi: 10.1038/s41591-018-0016-8 PMC6613387 29713087

[6] Pasqualucci L Dalla-Favera R. Genetics of Diffuse Large B-Cell Lymphoma. Blood (2018) 131:2307–19. doi: 10.1182/blood-2017-11-764332 PMC5969374 29666115

[7] Dubois S Tesson B Mareschal S Viailly PJ Bohers E Ruminy P. Lymphoma Study Association, Refining Diffuse Large B-Cell Lymphoma Subgroups Using Integrated Analysis of Molecular Profiles. EBioMedicine (2019) 48:58–69. doi: 10.1016/j.ebiom.2019.09.034 31648986 PMC6838437

[8] Chen YW Hu XT Liang AC Au WY So CC Wong ML. High BCL6 Expression Predicts Better Prognosis, Independent of BCL6 Translocation Status, Translocation Partner, or BCL6-Deregulating Mutations, in Gastric Lymphoma. Blood (2006) 108:2373–83. doi: 10.1182/blood-2006-05-022517 16772602

[9] Huang S Nong L Wang W Liang L Zheng Y Liu J. Prognostic Impact of Diffuse Large B-Cell Lymphoma With Extra Copies of MYC, BCL2 and/or BCL6: Comparison With Double/Triple Hit Lymphoma and Double Expressor Lymphoma. Diagn Pathol (2019) 14:81. doi: 10.1186/s13000-019-0856-7 31315646 PMC6637540

[10] Wight JC Chong G Grigg AP Hawkes EA. Prognostication of Diffuse Large B-Cell Lymphoma in the Molecular Era: Moving Beyond the IPI. Blood Rev (2018) 32:400–15. doi: 10.1016/j.blre.2018.03.005 29605154

[11] Kotlov N Bagaev A Revuelta MV Phillip JM Cacciapuoti MT Antysheva Z. Clinical and Biological Subtypes of B-Cell Lymphoma Revealed by Microenvironmental Signatures. Cancer Discov (2021) 11(6):1468–89. doi: 10.1158/2159-8290.CD-20-0839 PMC8178179 33541860

[12] Lenz G Wright G Dave SS Xiao W Powell J Zhao H. Stromal Gene Signatures in Large-B-Cell Lymphomas. New Engl J Med (2008) 359:2313–23. doi: 10.1056/NEJMoa0802885 PMC9103713 19038878

[13] Xu-Monette ZY Xiao M Au Q Padmanabhan R Xu B Hoe N. Immune Profiling and Quantitative Analysis Decipher the Clinical Role of Immune-Checkpoint Expression in the Tumor Immune Microenvironment of DLBCL. Cancer Immunol Res (2019) 7:644–57. doi: 10.1158/2326-6066.CIR-18-0439 30745366

[14] Cioroianu AI Stinga PI Sticlaru L Cioplea MD Nichita L Popp C. Tumor Microenvironment in Diffuse Large B-Cell Lymphoma: Role and Prognosis. Anal Cell Pathol (Amst) (2019) 2019:8586354. doi: 10.1155/2019/8586354 31934533 PMC6942707

[15] Ciavarella S Vegliante MC Fabbri M De Summa S Melle F Motta G. Dissection of DLBCL Microenvironment Provides a Gene Expression-Based Predictor of Survival Applicable to Formalin-Fixed Paraffin-Embedded Tissue. Ann Oncol (2018) 29:2363–70. doi: 10.1093/annonc/mdy450 PMC6311951 30307529

[16] Hopken UE Rehm A. Targeting the Tumor Microenvironment of Leukemia and Lymphoma. Trends Cancer (2019) 5:351–64. doi: 10.1016/j.trecan.2019.05.001 31208697

[17] Subramanian A Narayan R Corsello SM Peck DD Natoli TE Lu X. A Next Generation Connectivity Map: L1000 Platform and the First 1,000,000 Profiles. Cell (2017) 171:1437–1452.e17. doi: 10.1016/j.cell.2017.10.049 29195078 PMC5990023

[18] Kline J Godfrey J Ansell SM. The Immune Landscape and Response to Immune Checkpoint Blockade Therapy in Lymphoma. Blood (2020) 135:523–33. doi: 10.1182/blood.2019000847 31790142

[19] Xie M Huang X Ye X Qian W. Prognostic and Clinicopathological Significance of PD-1/PD-L1 Expression in the Tumor Microenvironment and Neoplastic Cells for Lymphoma. Int Immunopharmacol (2019) 77:105999. doi: 10.1016/j.intimp.2019.105999 31704289

[20] Mariathasan S Turley SJ Nickles D Castiglioni A Yuen K Wang Y. TGFβ Attenuates Tumour Response to PD-L1 Blockade by Contributing to Exclusion of T Cells. Nature (2018) 554:544–8. doi: 10.1038/nature25501 PMC6028240 29443960

[21] Hu J Xu J Yu M Gao Y Liu R Zhou H. An Integrated Prognosis Model of Pharmacogenomic Gene Signature and Clinical Information for Diffuse Large B-Cell Lymphoma Patients Following CHOP-Like Chemotherapy. J Transl Med (2020) 18:144. doi: 10.1186/s12967-020-02311-1 32228625 PMC7106727

[22] Cui Y Guo W Li Y Shi J Ma S Guan F. Pan-Cancer Analysis Identifies ESM1 as a Novel Oncogene for Esophageal Cancer. Esophagus (2021) 18:326–38. doi: 10.1007/s10388-020-00796-9 33175267

[23] London M Gallo E. Critical Role of EphA3 in Cancer and Current State of EphA3 Drug Therapeutics. Mol Biol Rep (2020) 47:5523–33. doi: 10.1007/s11033-020-05571-8 32621117

[24] Wang Z Wang X Zou H Dai Z Feng S Zhang M. The Basic Characteristics of the Pentraxin Family and Their Functions in Tumor Progression. Front Immunol (2020) 11:1757. doi: 10.3389/fimmu.2020.01757 33013829 PMC7461825

[25] Doni A Stravalaci M Inforzato A Magrini E Mantovani A Garlanda C. The Long Pentraxin PTX3 as a Link Between Innate Immunity, Tissue Remodeling, and Cancer. Front Immunol (2019) 10:712. doi: 10.3389/fimmu.2019.00712 31019517 PMC6459138

[26] Mirlekar B Pylayeva-Gupta Y. IL-12 Family Cytokines in Cancer and Immunotherapy. Cancers (Basel) (2021) 13(2):167. doi: 10.3390/cancers13020167 PMC7825035 33418929

[27] Larousserie F Kebe D Huynh T Audebourg A Tamburini J Terris B. Evidence for IL-35 Expression in Diffuse Large B-Cell Lymphoma and Impact on the Patient’s Prognosis. Front Oncol (2019) 9:563. doi: 10.3389/fonc.2019.00563 31316915 PMC6611226

[28] Zhou M Zhao H Xu W Bao S Cheng L Sun J. Discovery and Validation of Immune-Associated Long Non-Coding RNA Biomarkers Associated With Clinically Molecular Subtype and Prognosis in Diffuse Large B Cell Lymphoma. Mol Cancer (2017) 16:16. doi: 10.1186/s12943-017-0580-4 28103885 PMC5248456

[29] Bailey MH Tokheim C Porta-Pardo E Sengupta S Bertrand D Weerasinghe A. Comprehensive Characterization of Cancer Driver Genes and Mutations. Cell (2018) 173:371–85.e18. doi: 10.1016/j.cell.2018.07.034 29625053 PMC6029450

[30] Saffie R Zhou N Rolland D Onder O Basrur V Campbell S. FBXW7 Triggers Degradation of KMT2D to Favor Growth of Diffuse Large B-Cell Lymphoma Cells. Cancer Res (2020) 80:2498–511. doi: 10.1158/0008-5472.CAN-19-2247 PMC7417195 32350066

[31] Takeuchi T Yamaguchi M Kobayashi K Miyazaki K Tawara I Imai H. MYD88, CD79B, and CARD11 Gene Mutations in CD5-Positive Diffuse Large B-Cell Lymphoma. Cancer (2017) 123:1166–73. doi: 10.1002/cncr.30404 27915469

[32] Visco C Tanasi I Quaglia FM Ferrarini I Fraenza C Krampera M. Oncogenic Mutations of MYD88 and CD79B in Diffuse Large B-Cell Lymphoma and Implications for Clinical Practice. Cancers (Basel) (2020) 12(10):2913. doi: 10.3390/cancers12102913 PMC7600909 33050534

[33] Singh M Jackson KJL Wang JJ Schofield P Field MA Koppstein D. Lymphoma Driver Mutations in the Pathogenic Evolution of an Iconic Human Autoantibody. Cell (2020) 180:878–94.e19. doi: 10.1016/j.cell.2020.01.029 32059783

[34] Kraan W Horlings HM van Keimpema M Schilder-Tol EJ Oud ME Scheepstra C. High Prevalence of Oncogenic MYD88 and CD79B Mutations in Diffuse Large B-Cell Lymphomas Presenting at Immune-Privileged Sites. Blood Cancer J (2013) 3:e139. doi: 10.1038/bcj.2013.28 24013661 PMC3789201

[35] Muris JJ Meijer CJ Cillessen SA Vos W Kummer JA Bladergroen BA. Prognostic Significance of Activated Cytotoxic T-Lymphocytes in Primary Nodal Diffuse Large B-Cell Lymphomas. Leukemia (2004) 18:589–96. doi: 10.1038/sj.leu.2403240 14712286

[36] Tamma R Ranieri G Ingravallo G Annese T Oranger A Gaudio F. Inflammatory Cells in Diffuse Large B Cell Lymphoma. J Clin Med (2020) 9(8):2418. doi: 10.3390/jcm9082418 PMC7463675 32731512

[37] Kim S Nam SJ Park C Kwon D Yim J Song SG. High Tumoral PD-L1 Expression and Low PD-1(+) or CD8(+) Tumor-Infiltrating Lymphocytes are Predictive of a Poor Prognosis in Primary Diffuse Large B-Cell Lymphoma of the Central Nervous System. Oncoimmunology (2019) 8:e1626653. doi: 10.1080/2162402X.2019.1626653 31428525 PMC6685509

[38] Peng F Qin Y Mu S Li J Ai L Hu Y. Prognostic Role of Regulatory T Cells in Lymphoma: A Systematic Review and Meta-Analysis. J Cancer Res Clin Oncol (2020) 146:3123–35. doi: 10.1007/s00432-020-03398-1 PMC11804321 32995955

[39] McCord R Bolen CR Koeppen H Kadel EE 3rd Oestergaard MZ Nielsen T. PD-L1 and Tumor-Associated Macrophages in *De Novo* DLBCL. Blood Adv (2019) 3:531–40. doi: 10.1182/bloodadvances.2018020602 PMC6391660 30770362

[40] Haro M Orsulic S. A Paradoxical Correlation of Cancer-Associated Fibroblasts With Survival Outcomes in B-Cell Lymphomas and Carcinomas. Front Cell Dev Biol (2018) 6:98. doi: 10.3389/fcell.2018.00098 30211161 PMC6120974

[41] Autio M Leivonen SK Bruck O Mustjoki S Jorgensen JM Karjalainen-Lindsberg ML. Immune Cell Constitution in the Tumor Microenvironment Predicts the Outcome in Diffuse Large B-Cell Lymphoma. Haematologica (2021) 106(3):718–29. doi: 10.3324/haematol.2019.243626 PMC7927991 32079690

[42] Chen BJ Dashnamoorthy R Galera P Makarenko V Chang H Ghosh S. The Immune Checkpoint Molecules PD-1, PD-L1, TIM-3 and LAG-3 in Diffuse Large B-Cell Lymphoma. Oncotarget (2019) 10:2030–40. doi: 10.18632/oncotarget.26771 PMC6459346 31007846

[43] Fu J Li K Zhang W Wan C Zhang J Jiang P. Large-Scale Public Data Reuse to Model Immunotherapy Response and Resistance. Genome Med (2020) 12:21. doi: 10.1186/s13073-020-0721-z 32102694 PMC7045518

[44] Xu-Monette ZY Zhou J Young KH. PD-1 Expression and Clinical PD-1 Blockade in B-Cell Lymphomas. Blood (2018) 131:68–83. doi: 10.1182/blood-2017-07-740993 29118007 PMC5755041

[45] Quan L Lan X Meng Y Guo X Guo Y Zhao L. BTLA Marks a Less Cytotoxic T-Cell Subset in Diffuse Large B-Cell Lymphoma With High Expression of Checkpoints. Exp Hematol (2018) 60:47–56.e1. doi: 10.1016/j.exphem.2018.01.003 29353075

[46] Colaprico A Silva TC Olsen C Garofano L Cava C Garolini D. TCGAbiolinks: An R/Bioconductor Package for Integrative Analysis of TCGA Data. Nucleic Acids Res (2016) 44:e71. doi: 10.1093/nar/gkv1507 26704973 PMC4856967

[47] Cardesa-Salzmann TM Colomo L Gutierrez G Chan WC Weisenburger D Climent F. High Microvessel Density Determines a Poor Outcome in Patients With Diffuse Large B-Cell Lymphoma Treated With Rituximab Plus Chemotherapy. Haematologica (2011) 96:996–1001. doi: 10.3324/haematol.2010.037408 21546504 PMC3128218

[48] Dubois S Viailly PJ Bohers E Bertrand P Ruminy P Marchand V. Biological and Clinical Relevance of Associated Genomic Alterations in MYD88 L265P and Non-L265P-Mutated Diffuse Large B-Cell Lymphoma: Analysis of 361 Cases. Clin Cancer Res (2017) 23:2232–44. doi: 10.1158/1078-0432.CCR-16-1922 27923841

[49] Sha C Barrans S Cucco F Bentley MA Care MA Cummin T. Molecular High-Grade B-Cell Lymphoma: Defining a Poor-Risk Group That Requires Different Approaches to Therapy. J Clin Oncol: Off J Am Soc Clin Oncol (2019) 37:202–12. doi: 10.1200/JCO.18.01314 PMC6338391 30523719

[50] Godec J Tan Y Liberzon A Tamayo P Bhattacharya S Butte AJ. Compendium of Immune Signatures Identifies Conserved and Species-Specific Biology in Response to Inflammation. Immunity (2016) 44:194–206. doi: 10.1016/j.immuni.2015.12.006 26795250 PMC5330663

[51] Leek JT Johnson W Parker H Jaffe A Storey JJD. The SVA Package for Removing Batch Effects and Other Unwanted Variation in High-Throughput Experiments. Bioinformatics (2012) 28(6):882–3. doi: 10.1093/bioinformatics/bts034 PMC3307112 22257669

[52] Groth D Hartmann S Klie S Selbig J. Principal Components Analysis. Methods Mol Biol (Clifton NJ) (2013) 930:527–47. doi: 10.1007/978-1-62703-059-5_22 23086856

[53] Risso D Schwartz K Sherlock G Dudoit S. GC-Content Normalization for RNA-Seq Data. BMC Bioinf (2011) 12:480. doi: 10.1186/1471-2105-12-480 PMC3315510 22177264

[54] Zeng D Ye Z Yu G Wu J Xiong Y Zhou R. IOBR: Multi-Omics Immuno-Oncology Biological Research to Decode Tumor Microenvironment and Signatures. Front Immunol (2020) 12:687975. doi: 10.3389/fimmu.2021.687975 PMC8283787 34276676

[55] Howe KL Achuthan P Allen J Allen J Alvarez-Jarreta J Amode MR. Ensembl 2021. Nucleic Acids Res (2021) 490:D884–91. doi: 10.1093/nar/gkaa942 PMC7778975 33137190

[56] Love MI Huber W Anders S. Moderated Estimation of Fold Change and Dispersion for RNA-Seq Data With Deseq2. Genome Biol (2014) 15:550. doi: 10.1186/s13059-014-0550-8 25516281 PMC4302049

[57] Yu G Wang LG Han Y He QY. Clusterprofiler: An R Package for Comparing Biological Themes Among Gene Clusters. Omics: J Integr Biol (2012) 16:284–7. doi: 10.1089/omi.2011.0118 PMC3339379 22455463

[58] The Gene Ontology Consortium. The Gene Ontology Resource: 20 Years and Still GOing Strong. Nucleic Acids Res (2019) 47:D330–8. doi: 10.1093/nar/gky1055 30395331 PMC6323945

[59] Ogata H Goto S Sato K Fujibuchi W Bono H Kanehisa M. KEGG: Kyoto Encyclopedia of Genes and Genomes. Nucleic Acids Res (1999) 27:29–34. doi: 10.1093/nar/27.1.29 9847135 PMC148090

[60] Fabregat A Jupe S Matthews L Sidiropoulos K Gillespie M Garapati P. The Reactome Pathway Knowledgebase. Nucleic Acids Res (2018) 46:D649–55. doi: 10.1093/nar/gkx1132 29145629 PMC5753187

[61] Hänzelmann S Castelo R Guinney J. GSVA: Gene Set Variation Analysis for Microarray and RNA-Seq Data. BMC Bioinf (2013) 14:7. doi: 10.1186/1471-2105-14-7 PMC3618321 23323831

[62] Zhang B Horvath S. A General Framework for Weighted Gene Co-Expression Network Analysis. Stat Appl Genet Mol Biol (2005) 4:Article17. doi: 10.2202/1544-6115.1128 16646834

[63] Langfelder P Horvath S. WGCNA: An R Package for Weighted Correlation Network Analysis. BMC Bioinf (2008) 9:559. doi: 10.1186/1471-2105-9-559 PMC2631488 19114008

[64] Friedman J Hastie T Tibshirani R. Regularization Paths for Generalized Linear Models *via* Coordinate Descent. J Stat Softw (2010) 33:1–22. doi: 10.18637/jss.v033.i01 20808728 PMC2929880

[65] Yamaoka K Nakagawa T Uno T. Application of Akaike’s Information Criterion (AIC) in the Evaluation of Linear Pharmacokinetic Equations. J Pharmacokinet Biopharma (1978) 6:165–75. doi: 10.1007/BF01117450 671222

[66] Heagerty PJ Lumley T Pepe MS. Time-Dependent ROC Curves for Censored Survival Data and a Diagnostic Marker. Biometrics (2000) 56:337–44. doi: 10.1111/j.0006-341X.2000.00337.x 10877287

[67] Steyerberg EW Vickers AJ Cook NR Gerds T Gonen M Obuchowski N. Assessing the Performance of Prediction Models: A Framework for Traditional and Novel Measures. Epidemiol (Cambridge Mass) (2010) 21:128–38. doi: 10.1097/EDE.0b013e3181c30fb2 PMC3575184 20010215

[68] Schröder MS Culhane AC Quackenbush J Haibe-Kains B. Survcomp: An R/Bioconductor Package for Performance Assessment and Comparison of Survival Models. Bioinf (Oxford England) (2011) 27:3206–8. doi: 10.1093/bioinformatics/btr511 PMC3208391 21903630

[69] Harrell FE Jr. Regression Modeling Strategies: With Applications to Linear Models, Logistic and Ordinal Regression, and Survival Analysis. Cham: Springer (2015). doi: 10.1007/978-3-319-19425-7

[70] Tataranni T Piccoli C. Dichloroacetate (DCA) and Cancer: An Overview Towards Clinical Applications. Oxid Med Cell Longevity (2019) 2019:8201079. doi: 10.1155/2019/8201079 PMC6885244 31827705

[71] Kerr KF Brown MD Zhu K Janes H. Assessing the Clinical Impact of Risk Prediction Models With Decision Curves: Guidance for Correct Interpretation and Appropriate Use. J Clin Oncol: Off J Am Soc Clin Oncol (2016) 34:2534–40. doi: 10.1200/JCO.2015.65.5654 PMC4962736 27247223

[72] van der Maaten L. Accelerating T-SNE Using Tree-Based Algorithm. J Mach Learn Res (2014) 15:3221–45. doi: 10.5555/2627435.2697068

[73] Ligges U Mächler M. scatterplot3d - An R Package for Visualizing Multivariate Data. J Stat Softw (2003) 8(11):1–20. doi: 10.18637/jss.v008.i11

[74] Mayakonda A Lin DC Assenov Y Plass C Koeffler HP. Maftools: Efficient and Comprehensive Analysis of Somatic Variants in Cancer. Genome Res (2018) 28:1747–56. doi: 10.1101/gr.239244.118 PMC6211645 30341162

[75] Szklarczyk D Gable AL Lyon D Junge A Wyder S Huerta-Cepas J. STRING V11: Protein-Protein Association Networks With Increased Coverage, Supporting Functional Discovery in Genome-Wide Experimental Datasets. Nucleic Acids Res (2019) 47:D607–d613. doi: 10.1093/nar/gky1131 30476243 PMC6323986

[76] Shannon P Markiel A Ozier O Baliga NS Wang JT Ramage D. Cytoscape: A Software Environment for Integrated Models of Biomolecular Interaction Networks. Genome Res (2003) 13:2498–504. doi: 10.1101/gr.1239303 PMC403769 14597658

[77] Bader GD Hogue CW. An Automated Method for Finding Molecular Complexes in Large Protein Interaction Networks. BMC Bioinf (2003) 4:2. doi: 10.1186/1471-2105-4-2 PMC149346 12525261

[78] Chin CH Chen SH Wu HH Ho CW Ko MT Lin CY. cytoHubba: Identifying Hub Objects and Sub-Networks From Complex Interactome. BMC Syst Biol (2014) 8 Suppl 4:S11. doi: 10.1186/1752-0509-8-S4-S11 25521941 PMC4290687

[79] Han H Cho JW Lee S Yun A Kim H Bae D. TRRUST V2: An Expanded Reference Database of Human and Mouse Transcriptional Regulatory Interactions. Nucleic Acids Res (2018) 46:D380–6. doi: 10.1093/nar/gkx1013 29087512 PMC5753191

[80] Newman AM Liu CL Green MR Gentles AJ Feng W Xu Y. Robust Enumeration of Cell Subsets From Tissue Expression Profiles. Nat Methods (2015) 12:453–7. doi: 10.1038/nmeth.3337 PMC4739640 25822800

[81] Becht E Giraldo NA Lacroix L Buttard B Elarouci N Petitprez F. Estimating the Population Abundance of Tissue-Infiltrating Immune and Stromal Cell Populations Using Gene Expression. Genome Biol (2016) 17:218. doi: 10.1186/s13059-016-1070-5 27765066 PMC5073889

[82] Aran D Hu Z Butte AJ. xCell: Digitally Portraying the Tissue Cellular Heterogeneity Landscape. Genome Biol (2017) 18:220. doi: 10.1186/s13059-017-1349-1 29141660 PMC5688663

[83] Wilkerson MD Hayes DNJB. ConsensusClusterPlus: A Class Discovery Tool With Confidence Assessments and Item Tracking. Bioinformatics (2010) 26:1572–3. doi: 10.1093/bioinformatics/btq170 PMC2881355 20427518

[84] Wickham H. ggplot2: Elegant Graphics for Data Analysis. New York: Springer-Verlag (2016). doi: 10.1007/978-0-387-98141-3

[85] Patil I. Visualizations With Statistical Details: The’ggstatsplot’approach. J Open Source Softw (2021) 6(61). doi: 10.21105/joss.03167

