# Supplementary figures and images for: International Prognostic Index-Based Immune Prognostic Model for Diffuse Large B-Cell Lymphoma
Source: Front Immunol. 2021 Oct 22;12:732006. doi: 10.3389/fimmu.2021.732006 (PMC8569825; doi:10.3389/fimmu.2021.732006)

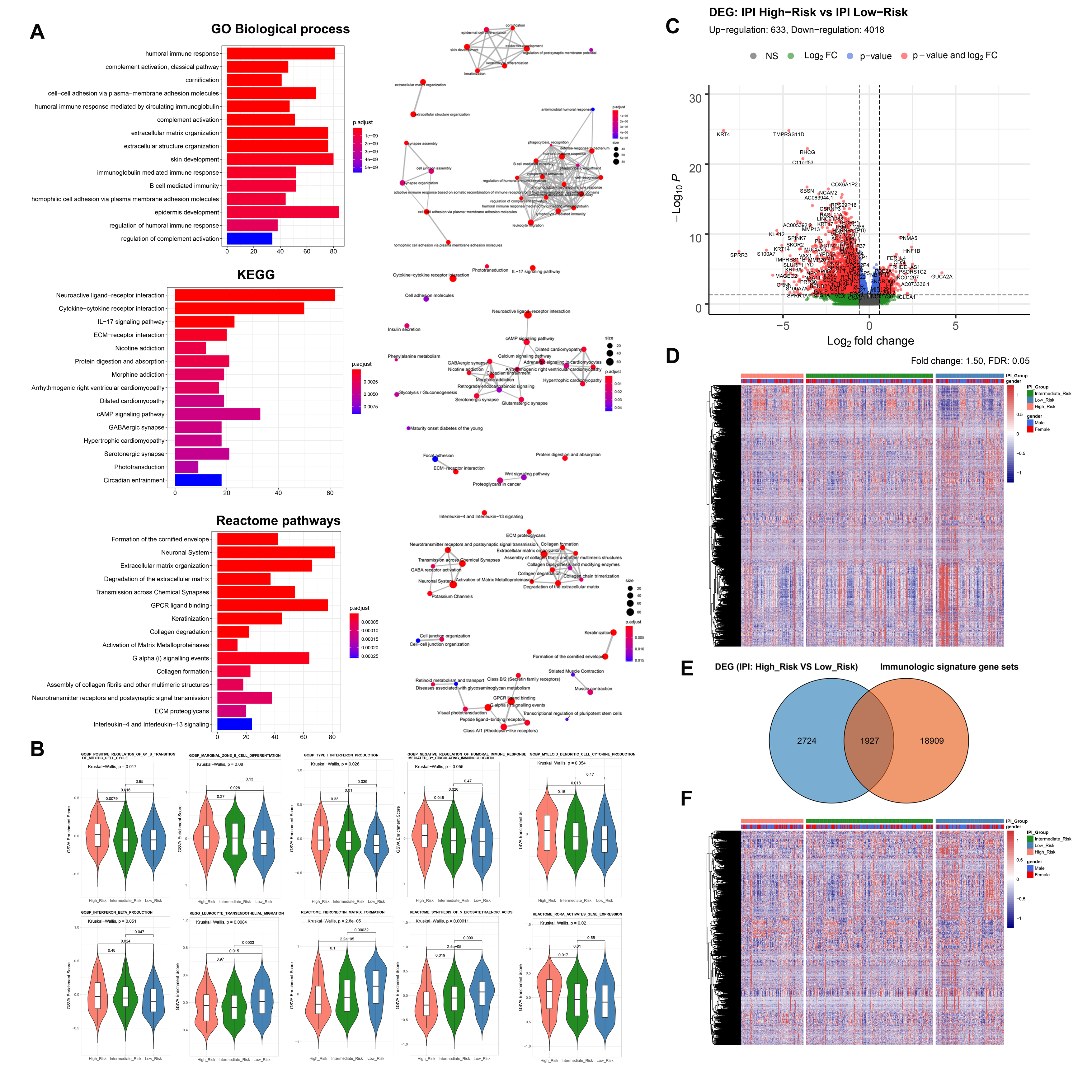

Supplement: Supplementary Figure 1 — Identification of DEGs of different IPI risk groups and gene functional enrichment analysis. (A) Volcano plot of differentially expressed genes (DEGs) between the high and low IPI Risk groups. (B) Heatmap of differentially expressed genes (DEGs) between the high and low IPI Risk groups. (C) Venn plot of intersection of DEGs and immune-related gene sets. (D) Heatmap of differentially expressed immune-related genes (DEGs) between the high and low IPI Risk groups. (E) Over representative analysis of the differentially expressed immune-related genes on GO biological process, KEGG and Reactome Pathway terms. (F) Comparation of GSVA results of IPI Risk groups on immune-related pathways. [file Image_1.tif]

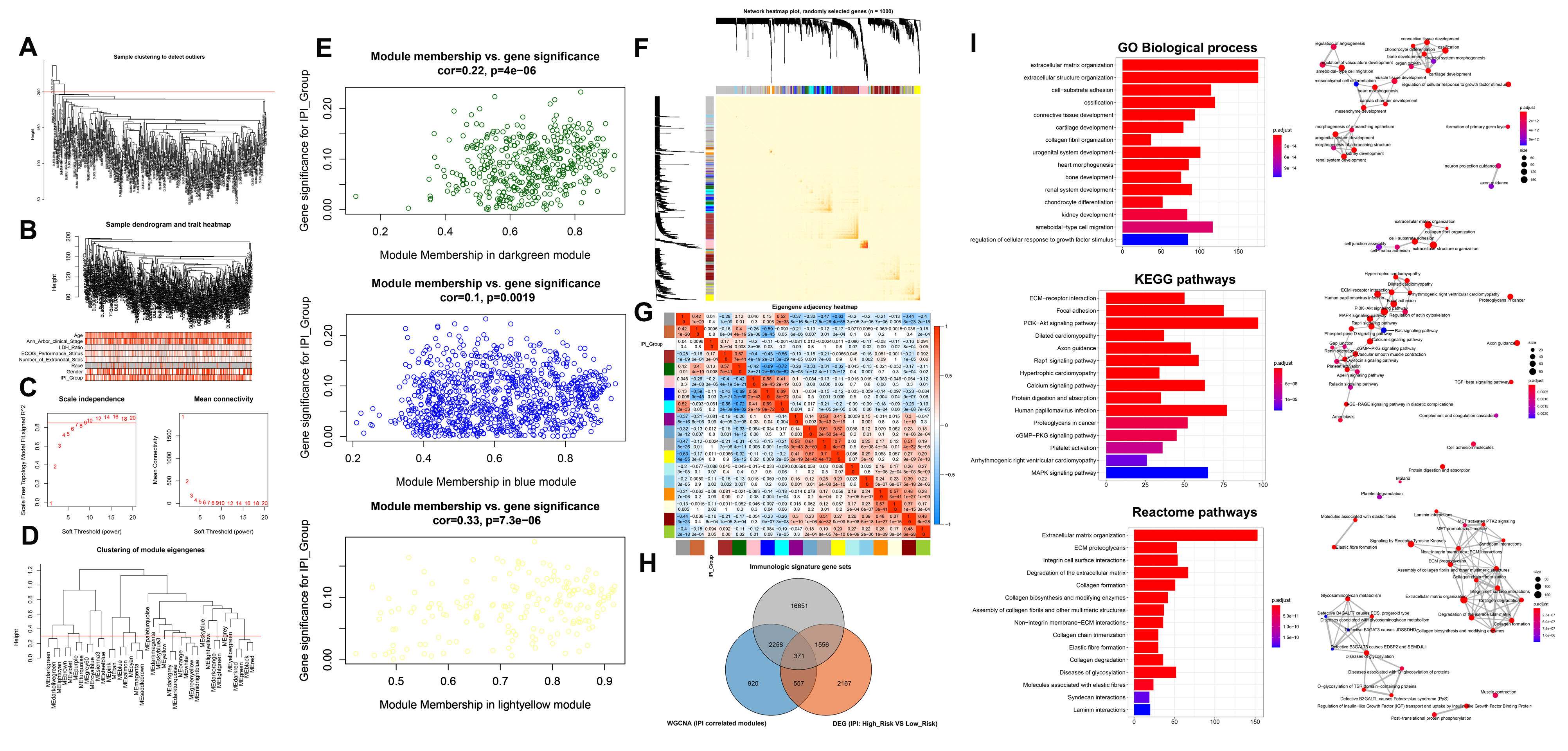

Supplement: Supplementary Figure 2 — WGCNA for the identification of modules related to IPI risk group and gene functional enrichment analysis for module genes. (A–D) Process of sample clustering and outlier detection, sample dendrogram, calculation of Beta value and soft thresholding, and clustering of module eigengenes. (E) Correlation between gene module membership and gene significance for IPI risk group in Darkgreen, Blue and Lightyellow modules. (F, G) Heatmaps of the Topological Overlap Matrix (TOM) and Eigengene adjacency. (H) Venn plot of intersection of IPI related module genes identified by WGCNA and immune-related gene sets. (I) Over representative analysis of the intersected module genes on GO biological process, KEGG and Reactome Pathway terms. [file Image_2.tif]

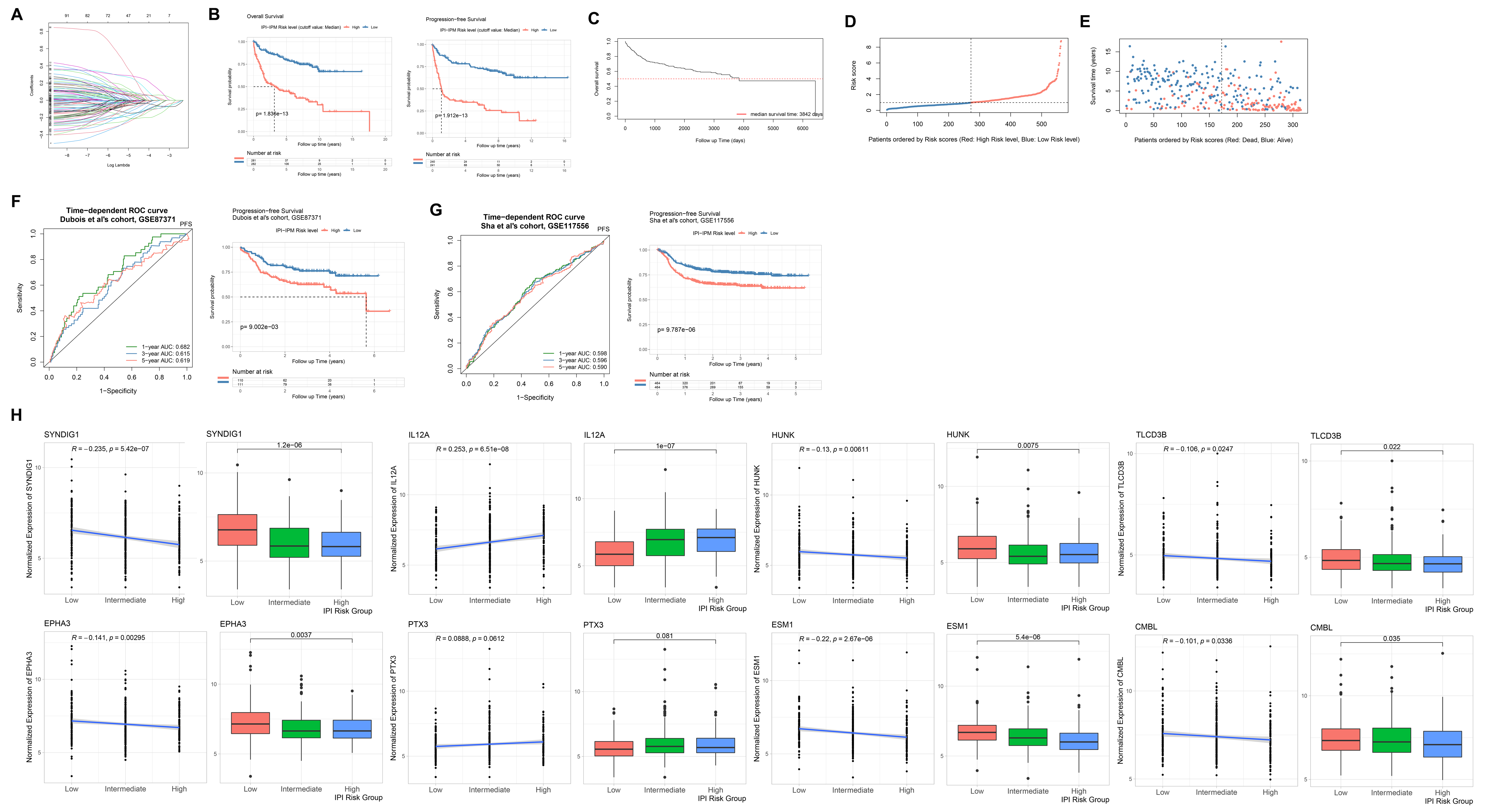

Supplement: Supplementary Figure 3 — Construction and validation of an IPI-based immune prognostic model. (A) Plot for the estimated coefficients with respect to the change of the penalty parameter of the Lasso penalized Cox regression. (B) Kaplan-Meier Survival analysis of OS and PFS for patients of high and low IPI-IPM risk groups (median as cutoff point). (C) Plot for median survival time of training cohort. (D and E) Risk scores and survival status of patients in high and low IPI-IPM risk groups. (F and G) Time-dependent ROC curve analysis of the IPI-IPM risk scores and Kaplan-Meier Survival analysis for patients of IPI-IPM high and low risk groups on PFS in validation cohorts (F: GSE87371 and G GSE117556). (H) Spearman correlation analysis of gene expression and IPI Risk group and difference test of gene expression between high and low IPI Risk group for the eight genes. [file Image_3.tif]

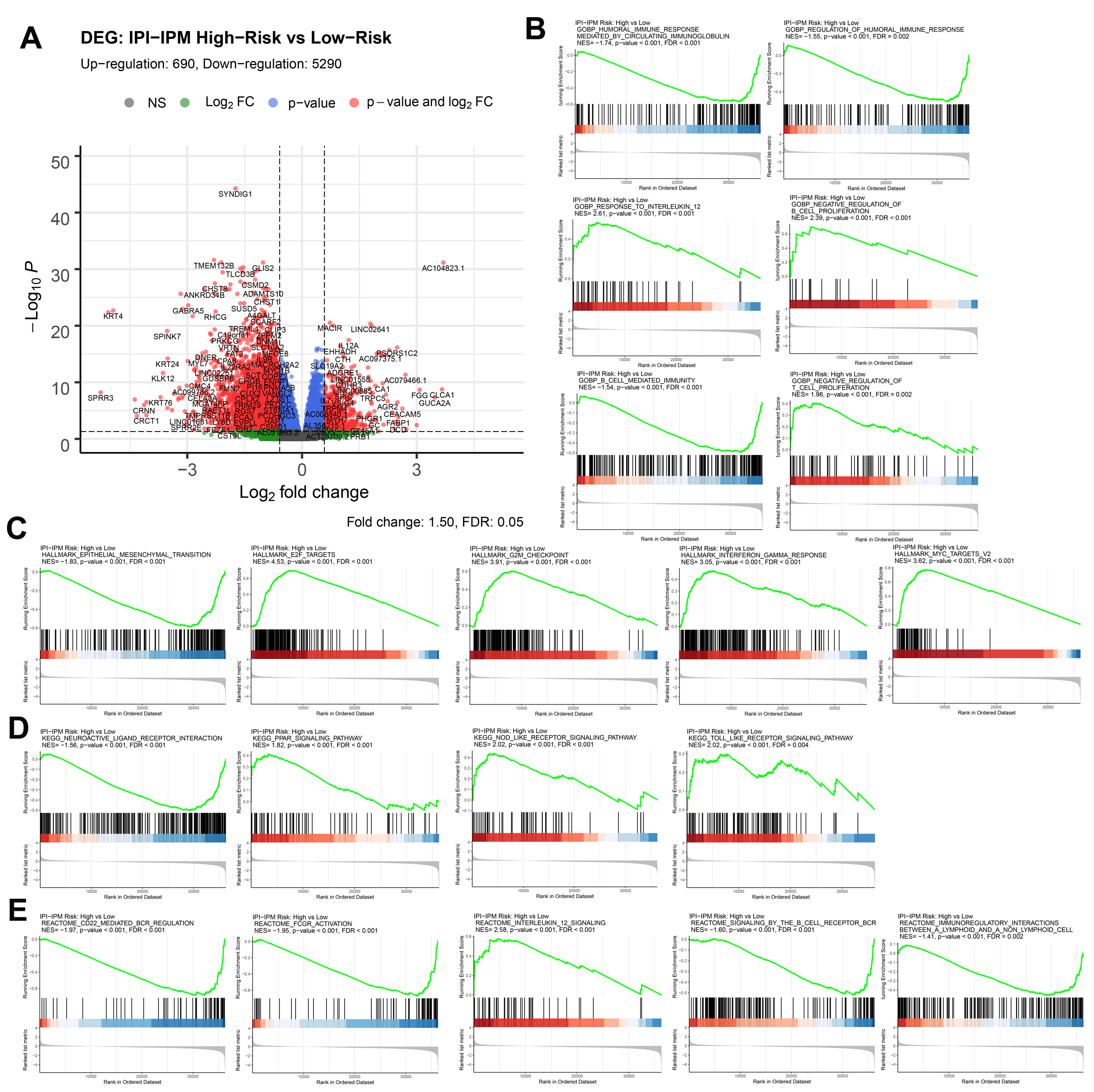

Supplement: Supplementary Figure 4 — Gene expression analysis of IPI-IPM and gene functional enrichment analysis. (A) Volcano plot of DEGs between high and low IPI-IPM risk group. (B–E) Pre-ranked GSEA of enriched gene sets between high and low IPI-IPM risk group on GO biological process, Hallmark gene sets, KEGG and Reactome Pathway terms. [file Image_4.tif]

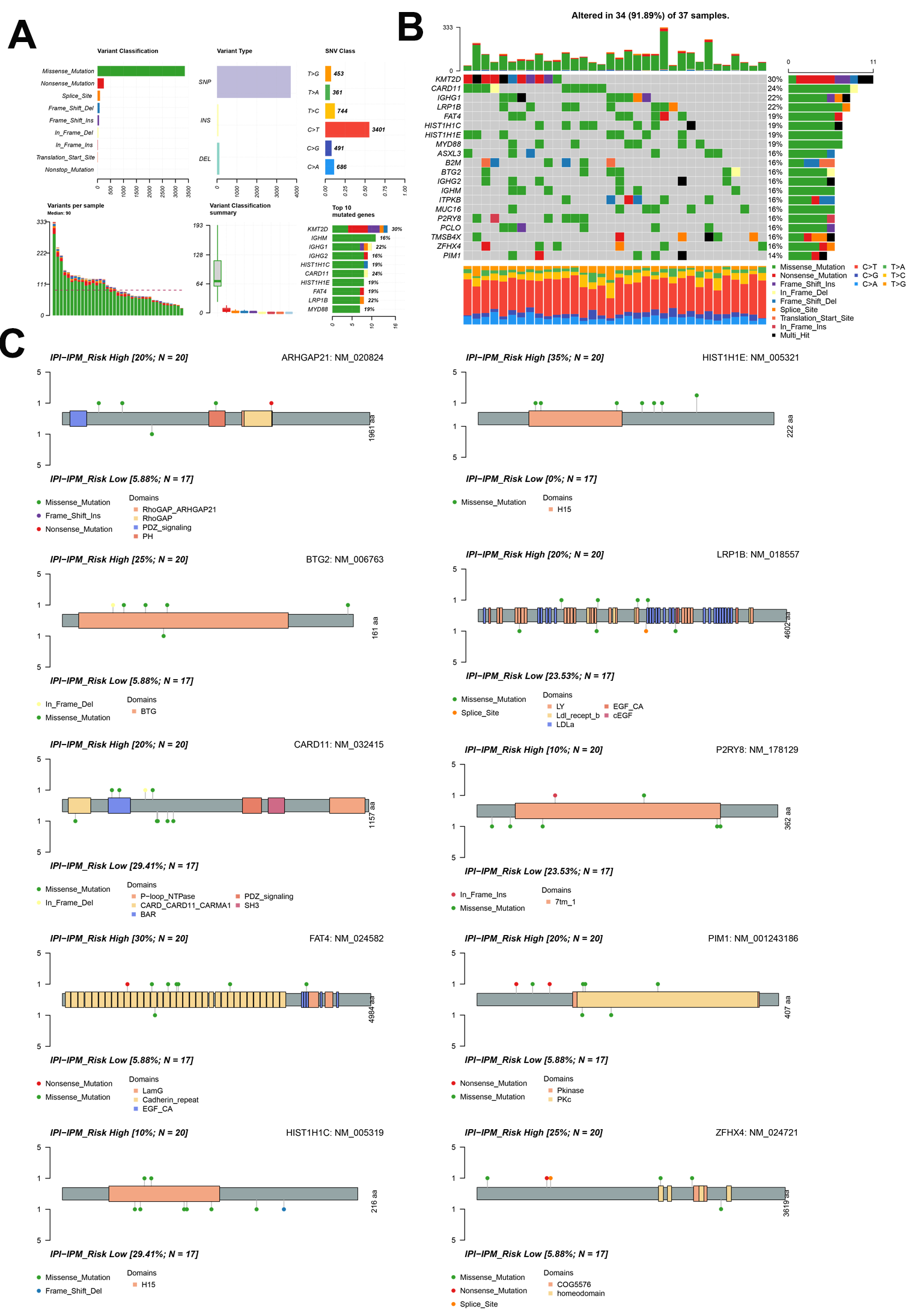

Supplement: Supplementary Figure 5 — Somatic mutational profiles of IPI-IPM subgroups. (A and B) SNV landscape and top mutated genes of all included samples. (B) Lollipop plots for amino acid changes of selected genes. [file Image_5.tif]

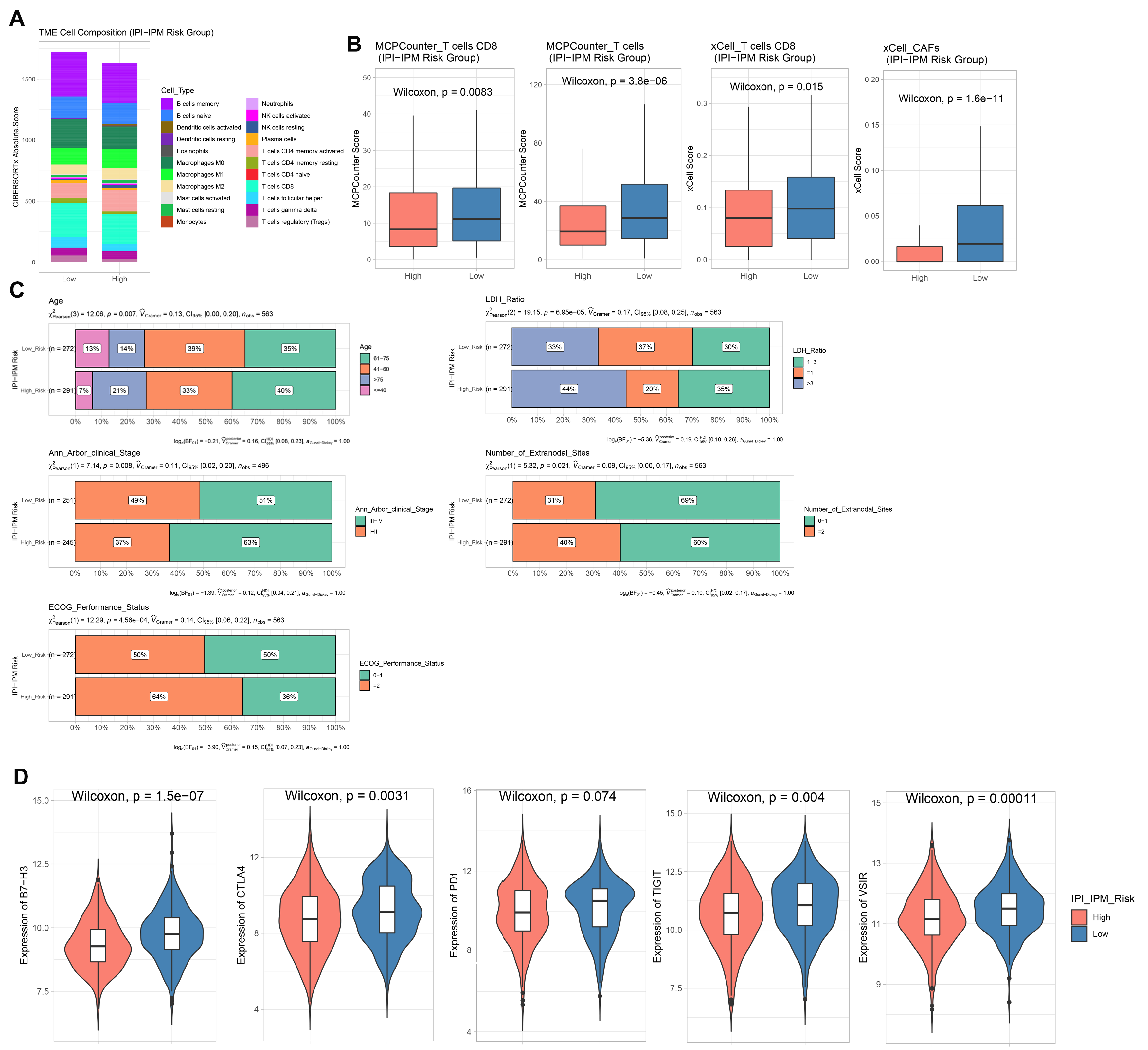

Supplement: Supplementary Figure 6 — TME characteristics, clinicopathological features and expression of inhibitory immune checkpoints in IPI-IPM subgroups. (A) Composition of immune cells infiltration by using the CIBERSORT algorithm. (B) Analysis of specific types of infiltrating immune cells by using the MCPcounter and xCell algorithm. (C) Relation of IPI-IPM Risk group and DLBCL clinicopathological features. (D) The expression of multiple inhibitory immune checkpoints between high and low IPI-IPM risk groups. [file Image_6.tif]
